# Three Linked Vasculopathic Processes Characterize Kawasaki Disease: A Light and Transmission Electron Microscopic Study

**DOI:** 10.1371/journal.pone.0038998

**Published:** 2012-06-18

**Authors:** Jan Marc Orenstein, Stanford T. Shulman, Linda M. Fox, Susan C. Baker, Masato Takahashi, Tricia R. Bhatti, Pierre A. Russo, Gary W. Mierau, Jean Pierre de Chadarévian, Elizabeth J. Perlman, Cynthia Trevenen, Alexandre T. Rotta, Mitra B. Kalelkar, Anne H. Rowley

**Affiliations:** 1 Department of Pathology, George Washington University School of Medicine, Washington, District of Columbia, United States of America; 2 Department of Pediatrics, Feinberg School of Medicine, Northwestern University, The Children’s Memorial Hospital, Chicago, Illinois, United States of America; 3 Department of Pathology, Feinberg School of Medicine, Northwestern University, The Children’s Memorial Hospital, Chicago, Illinois, United States of America; 4 Department of Microbiology/Immunology, Feinberg School of Medicine, Northwestern University, The Children’s Memorial Hospital, Chicago, Illinois, United States of America; 5 Department of Pathology, Stritch School of Medicine, Loyola University, Maywood, Illinois, United States of America; 6 Department Microbiology/Immunology, Stritch School of Medicine, Loyola University, Maywood, Illinois, United States of America; 7 Department of Pediatrics, School of Medicine, University of Southern California, Los Angeles Children’s Hospital, Los Angeles, California, United States of America; 8 Department of Pathology and Laboratory Medicine, The Children’s Hospital of Philadelphia, Philadelphia, Pennsylvania, United States of America; 9 Department of Pathology, Children’s Hospital Colorado, Aurora, Colorado, United States of America; 10 Department of Pathology and Laboratory Medicine, College of Medicine, Drexel University, St. Christopher’s Hospital for Children, Philadelphia, Pennsylvania, United States of America; 11 Department of Pathology and Laboratory Medicine, Alberta Children’s Hospital, Calgary, Alberta, Canada; 12 Department of Pediatrics, Riley Hospital for Children at Indiana University Health, Indianapolis, Indiana, United States of America; 13 Office of the Medical Examiner, Cook County Institute of Forensic Medicine, Chicago, Illinois, United States of America; S.G.Battista Hospital, Italy

## Abstract

**Background:**

Kawasaki disease is recognized as the most common cause of acquired heart disease in children in the developed world. Clinical, epidemiologic, and pathologic evidence supports an infectious agent, likely entering through the lung. Pathologic studies proposing an acute coronary arteritis followed by healing fail to account for the complex vasculopathy and clinical course.

**Methodology/Principal Findings:**

Specimens from 32 autopsies, 8 cardiac transplants, and an excised coronary aneurysm were studied by light (n=41) and transmission electron microscopy (n=7). Three characteristic vasculopathic processes were identified in coronary (CA) and non-coronary arteries: acute self-limited necrotizing arteritis (NA), subacute/chronic (SA/C) vasculitis, and luminal myofibroblastic proliferation (LMP). NA is a synchronous neutrophilic process of the endothelium, beginning and ending within the first two weeks of fever onset, and progressively destroying the wall into the adventitia causing saccular aneurysms, which can thrombose or rupture. SA/C vasculitis is an asynchronous process that can commence within the first two weeks onward, starting in the adventitia/perivascular tissue and variably inflaming/damaging the wall during progression to the lumen. Besides fusiform and saccular aneurysms that can thrombose, SA/C vasculitis likely causes the transition of medial and adventitial smooth muscle cells (SMC) into classic myofibroblasts, which combined with their matrix products and inflammation create progressive stenosing luminal lesions (SA/C-LMP). Remote LMP apparently results from circulating factors. Veins, pulmonary arteries, and aorta can develop subclinical SA/C vasculitis and SA/C-LMP, but not NA. The earliest death (day 10) had both CA SA/C vasculitis and SA/C-LMP, and an “eosinophilic-type” myocarditis.

**Conclusions/Significance:**

NA is the only self-limiting process of the three, is responsible for the earliest morbidity/mortality, and is consistent with acute viral infection. SA/C vasculitis can begin as early as NA, but can occur/persist for months to years; LMP causes progressive arterial stenosis and thrombosis and is composed of unique SMC-derived pathologic myofibroblasts.

## Introduction

Kawasaki disease (KD) is virtually as enigmatic today as it was when first reported as “mucocutaneous lymph node syndrome” in Japan in 1967 [1]. It is likely not a new disease, since subsequent studies demonstrated that cases diagnosed as “infantile peri-arteritis nodosa” were actually KD [2], [3]. It is referred to as a “self-limited acute vasculitis”, which predominantly strikes children three months to five years of age. It is especially common in Japan, where 1% of children develop the disease by age 5 [4], but it is also common in other Asian countries. With control of rheumatic heart disease, KD is recognized as the most common acquired childhood heart disease in developed nations. However, the true worldwide incidence, morbidity, and mortality will remain unknown until a diagnostic test is developed.

Although after 45 years, the exact etiology of KD remains unknown, the clinical, epidemiologic, and pathologic evidence supports a ubiquitous, possibly “new” infectious agent that likely causes only an inconsequential pulmonary infection, except in those children with a genetic predisposition for KD [5]. The respiratory prodromal infection appears to be due to an RNA virus, which creates persistent intracytoplasmic inclusion bodies in ciliated bronchial epithelium, and likely disseminates via macrophages to target medium sized arteries, most critically the coronaries [6]. The intracytoplasmic inclusion bodies manifest amphophilic staining by H&E (suggesting the presence of both protein and nucleic acid) and stain for RNA, but not DNA [6]. TEM of bronchial epithelium from three patients revealed virus-like particles associated with the intracytoplasmic inclusion bodies [7].

Although timely treatment with intravenous gamma globulin (IVIG) plus aspirin has profoundly reduced the prevalence of coronary artery damage [8], [9], [10], approximately 10–20% of patients fail therapy [11], [12], [13], [14], [15], [16] and are at an increased risk of developing coronary arteriopathy. Misdiagnosed cases and children not treated in a timely fashion or at all, including those who present with incomplete signs and symptoms (“atypical KD”), represent a serious problem of unknown dimensions. Recent use of Z-scores based on body surface area calculations to determine normal coronary artery measurements has led to the realization that coronary artery dilation is likely more prevalent and potentially more severe than previously appreciated [17], [18]. Inexplicably, infants less than one year and children over 8 years of age apparently have the highest prevalence of coronary pathology [4]. In 2007–2008 alone, 59 Japanese children with KD developed giant coronary artery aneurysms (CAA), considered the most dangerous type of coronary artery (CA) pathology [4]. Therefore, both treated and untreated KD children still develop serious heart disease.

Early gross and light microscopic autopsy studies attempted to identify the characteristic CA histopathology, a chronology, and establish clinical:pathologic correlation. They were confounded by the complexity of the vasculitis, which typically varies from vessel to vessel of the same patient, among sections from the same vessel, and even around the circumference of a single section [19], [20], [21]. The initial studies described microvessel edema followed by a medium-sized arteritis comprised of neutrophils, lymphocytes, and large mononuclear cells progressing to panarteritis, and elastic lamina and smooth muscle cell (SMC) destruction leading to aneurysm formation. This pathology was observed in the first two months after KD onset and reported to be followed by resolution of the inflammation and fibrous scar formation, leading to CA stenosis in some cases [19], [20].

A 2010 review summarized what the authors described as “consistent among Japanese researchers”: “Firstly, the histological characteristic of vasculitis in Kawasaki disease is proliferative granulomatous inflammation consisting of markedly accumulating monocytes/macrophages”, “Secondly, vasculitis in Kawasaki disease starts simultaneously with the onset, rapidly reaches an inflammation peak, and then slowly remits and heals with cicatrization, showing a monophasic course.” It was stated that “the course of Kawasaki disease was synchronous throughout the body” [22].

We studied KD specimens from autopsies (n=32), explanted hearts (n=8), and an incidentally detected CAA resection by light (LM) and transmission electron microscopy (TEM), and identified a vasculopathic scheme unlike any previously described [19], [20], [22]. Our study identified three distinct but linked basic KD vascular processes: necrotizing arteritis (NA), subacute/chronic (SA/C) vasculitis, and luminal myofibroblastic proliferation (LMP).

## Methods

### Ethics Statement

The study was approved by the Institutional Review Board of Children’s Memorial Hospital and tissues were de-identified to maintain confidentiality.

### Patients and Tissues

The patients are described in Table 1; all specimens were studied by light microscopy. Seven cases studied by TEM were either retrieved from prolonged formalin fixation or from paraffin blocks. Cases were located when a family member contacted us, through discussion with colleagues, or occasionally via published reports. This collection represented a random sample of KD patients who died, received cardiac transplants, or in one case, underwent surgical excision of an asymptomatic coronary artery aneurysm. While two cases (1 and 6) had only a single available H&E-stained CA/myocardial section, most had multiple CA and organ sections, the original blocks from the autopsy or transplanted heart, or portions of the formalin-fixed autopsy tissue. Cases 4 [23], 17 and 36 [2], 21 and 22 [24], and 24 [25] were previously published.

**Table 1:** Demographic and clinical data on Kawasaki Disease patients in this study.

Case	Age	Time since onset (yr of specimen)	Sex	Ethnicity	KD Therapy	Specimen
1	3 yr	10 days (1970s)	U	Japanese	None	Autopsy-CA
2	10 yr	13 days (1994)	M	Caucasian	IVIG, A, W	Autopsy-CA
3	3 yr	2 wks (1995)	M	Caucasian	IVIG, A	Transplant
4	11 mo	2.5 wks (1997)	M	Caucasian	None	Autopsy
5	22 mo	2.5 wk (1976)	M	Caucasian	None	Autopsy
6	3 mo	2.5 wk (1974)	F	Caucasian	None	Autopsy-CA
7	4 mo	3 wk (2000)	M	Caucasian	IVIG, A	Autopsy
8	8 mo	3 wk (1995)	F	U	None	Autopsy
9	21 mo	3 wk (1985)	F	U	A, D, H	Autopsy
10	7 mo	3.5 wk (1978)	F	Caucasian	None	Autopsy
11	3.5 mo	3–4 wk (2006)	M	Caucasian	IVIG, A, S	Autopsy-TEM
12	4 mo	3–4 wk (1971)	M	Caucasian	None	Autopsy-CA
13	4.5 mo	4 wk (2008)	M	Hispanic	IVIG, A, I, S, H,W	Autopsy-TEM
14	4 mo	4 wk (1997)	M	Hispanic	None	Autopsy
15	7 mo	4 wk (1982)	F	Caucasian	None	Autopsy
16	10 mo	4 wk (1984)	F	AA	A, D	Autopsy
17	6 mo	4–5 wk (1974)	M	Caucasian	S	Autopsy
18	4 mo	5 wk (2005)	M	U	IVIG, A, S, H, W	Autopsy-CA
19	2 mo	5 wk (2001)	M	Caucasian	IVIG,A,S,H, ECMO	Autopsy
20	3.5 mo	6 wk (2002)	M	Japanese-American	None	Autopsy-CA
21	4 mo	6 wk (1986)	F	Caucasian	A, H	Autopsy
22	6 mo	6.5 wk (1997)	M	Asian	IVIG, A, S, C, D, H, W,T	Autopsy
23	4 mo	2 mo (1999)	F	Caucasian	IVIG, A, S, M, H	Autopsy
24	6 mo	3 mo (1993)	M	Caucasian	IVIG, A	Transplant
25	2 yr	4.5 mo (2009)	F	Greek	IVIG, A	Autopsy-CA
26	6 yr	5 mo (2008)	M	Caucasian	None	Transplant-TEM
27	5 yr	7.5 mo (2008)	F	Caucasian	IVIG, A, I, prolonged S	Autopsy-TEM
28	19 mo	10 mo (1998)	M	AA	None	Autopsy
29	5 yr	1 yr (2000)	M	Caucasian	None	Autopsy
30A	19 mo	15 mo (1994)	F	Caucasian	IVIG, A	Transplant 1-TEM
30B	12 yr	11.5 yr (2005)				Transplant 2
30C	16 yr	14 yr (2007)				Transplant 3-TEM
31	8 yr	16 mo (1993)	F	Asian-American	IVIG, A	Thoracic autopsy
32	2 yr	18 mo (1999)	M	Caucasian	A, W	Transplant-TEM
33	3 yr	2 yr (2000)	M	U	U	Transplant
34	19 yr	16 yr (2008)	M	Hispanic	None	CA excision-TEM
35	22 yr	19 yr	F	Hispanic	A	Autopsy
36	13 wk	U (1972)	F	Caucasian	U	Autopsy
37	14 mo	U (2002)	M	U	U	Transplant
38	17 mo	U (1997)	M	U	U	Transplant
39	11 yr	U (1999)	M	Caucasian	IVIG, A	Autopsy
40	11 mo	U (1954)	F	Caucasian	None	Autopsy
41	7 mo	U (1959)	F	Caucasian	None	Autopsy

CA=coronary arteries, TEM=transmission electron microscopy, IVIG=intravenous gammaglobulin, A=aspirin, AA=African-American, U=unknown, S=methylprednisolone/steroid, W=warfarin, H=heparin, D=dipyridamole, I=infliximab, ECMO=extracorporeal membrane oxygenation, C=cyclophosphamide, M=methotrexate, T=tissue plasminogen activator.

### Antibodies and Light Microscopy Stains

Histochemical staining and immunohistochemistry (IHC) were performed in a certified pathology department histology laboratory using standard techniques. Masson trichrome, elastin van-Gieson, macrophage (CD68), Factor VIII-related antigen, and alpha-smooth muscle actin (SMA) (Dako) stains were utilized with appropriate controls. Elastin van-Gieson highlights the internal (IEL) and external elastic (EEL) lamina, the fine elastin fibrils of the media, and the coarse fibers of endocardial fibroelastosis. SMA highlights medial and adventitial SMC and LMP myofibroblasts. Myofibroblasts often dually stained with trichrome; peripheral red for actin and central blue for the extracellular matrix (ECM) precursors within the rough endoplasmic reticulum (RER). The LMP ECM variably stained blue with the trichrome stain and pink with the H&E stain.

Depending on their availability, tissue/vessels and blocks were repeatedly re-sectioned and stained to determine the extent and features of the vascular involvement.

### TEM

Un-embedded formalin-fixed and paraffin-embedded tissue was trimmed for areas of greatest interest and cut into pieces of 1 mm or less for resin embedding. Retrieval from paraffin for TEM was performed as previously described [26]. Resin embedding routinely started with 8 blocks, and proceeded by multiples of 8, until adequate and desired sampling was achieved. Semi-thin, one micron resin LM sections were cut with glass knives and stained with our routine methylene blue/azure II and basic fuchsin “trichrome” stain [26]. Ultra-thin sections were cut with a diamond knife from blocks selected for TEM, contrasted with uranyl acetate and lead citrate, and examined with a LEO EM10 transmission electron microscope operating at 60 kV.

## Results

The earliest death in our series was in a child with CA vasculopathy and interstitial “eosinophilic-type” myocarditis (Case 1, day 10) (Table 2). The six deaths from ruptures of CAA were early, between days 13 and 21, due to saccular aneurysms caused by NA and composed of varying amounts of preserved adventitia only.

**Table 2:** Pathologic findings in patients with Kawasaki Disease.

Case	Time since onset	Cause of death/TX	CA pathology	Non-CA pathology	Myocardium	Comments
1	10 days	Myocarditis	SA/C-LMP	N/A	“Eosinophilic myocarditis”, marked edema, no necrosis or PMNL	Several slides from one block
2	13 days	Ruptured LAD CAA	Waning necrotizing arteritis, thrombosed. SA/C-LMP LAD	Intra-renal SA/C		Gallbladder hydrops
3	2 wks	Ruptured LCAA, TX	CABG & over-sewn hole, no SA/C- LMP visible	N/A	Acute hemorrhagic MI, PMNL. MI subacute, GT	Free wall atrophy
4	2.5 wk	Ruptured giant LAD CAA	Giant CAA LAD, x2 RCA. SA/C-LMP	N/A	Large acute MI, no PMNL	Acute pancreatitis and splenitis
5	2.5 wk	Ruptured left main CAA	RCAA thrombosed. SA/C-LMP	SA/C testicular artery	Acute MI, PMNL	Severe acute pancreatitis, adrenal medullary calcification, thymic atrophy
6	2.5 wk	MI	CAA RCA, LAD, thrombosed. SA/C-LMP LAD occluded	By report: SA/C-LMP, renal, peri-esophageal, mesenteric, peri-uterine, ovarian, peri-adrenal	Subacute MI, GT	One slide
7	3 wk	MI	Thrombosed CAA. SA/C-LMP	SA/C-LMP aortic, testicular	Acute MI, no PMNL or necrosis	Marked pericarditis, thymic necrosis, acute splenitis, TV valvulitis
8	3 wk	Ruptured CAA	CAA, fresh & organizing thrombi, calcified. SA/C-LMP	N/A	Edema, atrophy	Mild peri-ductal chronic pancreatitis. Few sections
9	3 wk	Ruptured giant LAD CAA	Fresh thrombi, LAD, LCx, RCAA	SA/C-LMP, iliacs.	Acute MI, PMNL	CAAs, long, sausage-shaped
10	3.5 wk	MI	RCAA fresh thrombus. LCA SA/C-LMP	SA/C-LMP, acute renal thrombosis	Pseudo-myocarditis	Thymic calcification, renal infarct
11	3–4 wk	Thrombosed mesenteric aneurysm, organizing, recanalized, SI infarct	Aneurysms, CAAs. Fresh thrombus RCAA. Severe SA/C-LMP	Thrombosed aneurysms, inter-costal, femoral, axillary, splenic. SA/C-LMP, hepatic, skin aortic, adrenal, renal, thymus. Ilial artery thrombosed, organizing, recanalizing	Extensive MI, PMNL, necrosis.	SA/C-LMP veins, Renal, adrenal infarcts, terminal ileum necrosis
12	3–4 wk	MI	CAAs fresh & organizing calcified thrombi. Long dilated thrombosed CAs with SA/C-LMP to 95%	N/A	Extensive subacute MI, GT. Propagating acute necrosis, no PMNL	
13	4 wk	Ruptured RCIAA	Multiple CAA, SA/C-LMP CAs	SA/C-LMP hepatic, renal, illiacs prostatic. Aneurysms CIAs, axillary	Mild interstitial fibrosis. No acute ischemia	Five distal aortic aneurysms up to 8 mm
14	4 wks	MI	CAAs, LAD, LCx thrombosed. SA/C-LMP, severe	Aortitis	Focal subacute MIs, GT	Viral-type pneumonia, heart failure
15	4 wk	Massive MI	CAA, LAD, RCA, thrombosed. SA/C, no LMP	SA/C-LMP cystic duct artery	Acute MI, PMNL. Mural thrombi. Mitral insufficiency	Gallbladder hydrops, chronic pancreatitis, central liver necrosis
16	4 wk	MI	CAAs, RCA, LCA, LAD, LCx, thrombosed	SA/C-LMP marked, all organs, stenotic with thrombi, aortitis	MI subacute, GT. Interstitial SA/C	Concurrent group B Salmonella sepsis. Most CAA and non-CA disease seen in our cases
17	4–5 wk	MI	CAAs, LAD, LCX, RCA, large fresh thrombi, SA/C-LMP	SA/C-LMP, epididymis, pancreas	Massive acute MI, odd-shaped, not confluent, no PMNL	Cholangitis, pericarditis, thymic involution
18	5 wk	MI	Acute thrombosis LCxCAA, RCAA, SA/C-LMP	N/A	Acute ischemia, no PMNL	Vavulitis
19	5 wk	MIs	Giant LAD CAA, fresh & organizing thrombi, calcified, recanalized, RCAA, SA/C-LMP, severe	SA/C-LMP, mesenteric, organizing thrombus. Mild LMP PA, IVC	Extensive non-confluent SA and healing MIs, calcifying	“Atypical myocytic process”. Generalized calcification, pigmented macrophages. Rt carotid thrombosis, collapse, S/P ECMO
20	6 wk	MI	CAA, fresh, organizing & organized thrombi	By report: Thrombosed inter-costal aneurysms. Thickened iliacs and aortic bifurcation	Acute ischemia, no PMNL	Five sections from one CA only
21	6 wk	MI	RCAA fresh thrombus. SA/C-LMP LAD & LtCx, organized thrombi	SA/C-LMP & thrombi, renal, pancreatic, mesenteric, muscle, iliac, hepatic	N/A	Acute renal, splenic infarcts.
22	6.5 wk	MI	CAA, fresh, organizing, organized thrombi, calcified	SA/C-LMP, SMA. LMP subclavian	Healing MI, GT, acute propagation, no PMNL	Necrotic adrenal medullae with macs. Cholangitis, sialadenitis, chronic enteritis. Splenic collections of macrophages
23	2 mo	MI	CAA, RCA, LAD, fresh, calcified, organizing thrombi, calcified	SA/C-LMP, renal, femoral, mesenteric, splenic	Acute MI, no PMNL. Sub-acute MI, GT	Renal, splenic infarcts. Hemorrhagic peritonitis
24	3 mo	MI; TX	CAAs, organizing, organized calcified thrombi. RCAA, long fresh thrombus. Focal SA/C-LMP	N/A	Acute MI, no PMNLs. Healed MI	TV valvulitis
25	4.5 mo	MI	CAA & SA/C-LMP	N/A	MI, minimal necrosis, no PMNL	
26	5 mo	MI;TX	RCAA no thrombi. Marked SA/C-LMP	N/A	Focal ischemia, no PMNL	Minimal epicarditis
27	7.5 mo	MI	Multiple CAA, no thrombi. Marked SA/C-LMP RCA, LCx, LAD, 90–100% stenosed	SA/C-LMP, pancreatic, splenic, renal, aortitis, uterine, >90% stenotic, paratracheal occluded	Acute necrosis, foci of PMNL. Calcified myocytes	Mild cerebral vessel lymphocyte infiltrate
28	10 mo	MI	SA/C-LMP to 95% stenotic, one fresh thrombus	SA/C-LMP, PA, aortitis with intimal multinucleated macrophages	Acute necrosis, PMNL. Interstitial fibrosis. Myocyte calcification	Valvulitis, PV, MV, TV. Pulmonary emboli
29	1 yr	MI	Giant CAA, organizing thrombus. SA/C-LMP LAD, 4 cm dilation	SA/C-LMP, renal, SI, adrenal, LN, hepatic, sklt muscle, PA, thyroid, stomach	Acute necrosis, mild PMNL. Rare foci of healing ischemia, GT	
30A	15 mo	MI; TX1	RCAA fresh & organized thrombus. LCAA organizing thrombus, calcified. SA/C-LMP	N/A	Acute ischemia, no PMNL	Hypertrophied, box-car nuclei
30B	11.5 yr	MI; TX2	CAs to 90% luminal stenosis	N/A	Massive necrosis, no PMNL. Mild fibrosis	Mild medial, IEL damage, IEL reduplicated. Rare intimal foamy macs, SA/C, prominent lymphocyte collections
30C	14 yr	CA insufficiency; TX3	Luminal occlusion LAD, RCA, LCx CA. Intimal foamy macs, SMC. Mast cells	N/A	No acute or chronic ischemia. Lymphocytic pseudo-myocarditis	SA/C-LMP veins. Preserved CA media, IEL
31	16 mo	MI	Lt main CAA thrombosed. SA/C-LMP, LCx, LAD, RCA	N/A	Mild ischemia, no PMNL. Healed foci of ischemia	Thorax only
32	18 mo	Chronic ischemia; TX	RCAA. SA/C-LMP, no thrombi	N/A	Massive scarring. No inflammation, acute ischemia	
33	2 yr	MI; TX	Lt main CAA, fresh & organizing thrombi, calcified	SA/C-LMP, variable stenosis	Large subacute MI, GT. Trapped coagulation necrosis, no PMNL	“Atypical myocytic process”
34	16 yr	Incidental finding during cardiac catheterization for WPW; aneurysm resected	RCAA. SA/C-LMP	N/A	N/A	IMA graft
35	19 yr	MI	SA/C-LMP to 80% stenotic	N/A	Acute MI with PMNL	
36	U	MI	Thrombosed CAA	No SA/C-LMP	Mild necrosis, PMNL Atrophy	SA/C, necrosis of MV. Limited slides
37	U	MI; TX	LADCAA, fresh thrombus. SA/C-LMP thrombi, organized, re-canalized, calcified	N/A	Healing and near-healed MI. Small acute MI, no PMNL	Prominent, generalized eosinophils
38	U	MI	Giant CAA LAD, fresh & organizing, thrombi, re-canalized, calcified. RCAA. SA/C-LMP to 90% stenosis	N/A	Massive acute necrosis, no PMNL. Large healing LV scars with elastosis	Reduplication of LCx IEL
39	U	MI	RCAA, long fresh thrombus, CAA thrombus, organized, calcified	N/A	Necrosis, no PMNL. Healed MI	TV valvulitis
40	U	MI	CAAs, fresh thrombi	By report: SA/C-LMP, hepatic, spermatic, retro-peritoneal	Hyper-eosinophilia, pseudo-myocarditis	By report: necrosis, thrombosis of choroid plexus, corpus striatum, calcified. Only heart slides
41	U	MI	Giant CAAs, fresh & organizing thrombi, calcified. SA/C-LMP	By report: SA/C-LMP, pancreatic, mesenteric, renal, peri-aortic, splenic, skeletal muscle	No acute changes. Stellate healing MIs, GT	Only heart slides

RCIA=right common iliac artery, SA/C=subacute/chronic inflammation, CA=coronary artery, CAA=coronary artery aneurysm, LMP=luminal myofibroblastic proliferation, MI=myocardial infarction, TX=transplant, Mac=macrophage, SI=small intestine, RCA=right coronary artery, LAD=left anterior descending artery, LCx=left circumflex artery, IMA=inferior mesenteric artery, GB=gallbladder, MV=mitral valve, TV=tricuspid valve, PV=pulmonary valve, GT=granulation tissue, eos=eosinophils, SMA=superior mesenteric artery, PA=pulmonary arteries, macs=macrophages, PMNL=predominantly neutrophils, U=unknown, N/A=not available, EFE=endocardial fibroelastosis, ECMO=extracorporeal membrane oxygenation, IVC=inferior vena cava. Notes: CA sections usually contained myocardium, epicardium, and some endocardium. All patients had active SA/C plus LMP in coronary and also in available non-coronary arteries. Varying degrees of chronic hypoxia (hydropic change) seen in all cases. Anitschkow cells always seen, if case had at least two sections of myocardium. EFE always seen, if specimens included more than focal endocardium. By report=slides not available, but description sufficient to derive pathology.

Myocardial infarction (MI) due to thrombosed saccular CAA was the most common cause of death, occurring in 23 patients; two additional patients received transplants for myocardial infarction due to thrombosis. The time from KD onset to MI was known for 19 of these 25 cases and ranged from 19 days to 2 years.

Six patients had MI due to stenosis from SA/C-LMP without detectable thrombi; three of these did not have saccular CAA. They occurred between 4.5 months and 19 years after onset, with 5 of 6 occurring within two years, similar to the timing of the MIs in the 19 patients with thrombosis of saccular CAA.

The other two deaths were due to a ruptured iliac aneurysm (case 13) and a thrombosed mesenteric artery (case 11). At autopsy, these patients also had severe CA disease that soon could have been critical. The final patient had an incidental CAA (case 34) resected at 16 years after onset.

Early into the study, it became clear that our observations did not readily conform to the previously described histopathology or chronology [19], [20], [21], [22]. Previously published light micrographs often demonstrated histopathology similar to what we observed, but the descriptions and interpretations differed markedly, leading to alternative conclusions. Our findings are most similar to those of Amano et al [27].

Three distinct basic vasculopathic processes were consistently demonstrated in the CA and medium-sized muscular and elastic non-coronary (NCA) arteries of our patients: NA, SA/C vasculitis, and LMP (Table 2). All three processes were present in the CA sections from the patient who died from a CAA rupture that occurred within the first two weeks after fever onset (Case 2, day 13). The first death (Case 1, day 10) was in a patient who had both “eosinophilic-type” interstitial myocarditis and CA SA/C inflammation with LMP; NA was not present in the several available sections from a single CA.

Fibrinoid necrosis was not observed in our material. However, layers of fibrin, which stained homogeneously eosinophilic (“hyaline”) by H&E and lacked necrotic debris, were observed and could be mistaken for fibrinoid necrosis.

Each case was evaluated as a challenge to the three process scheme, and all were confirmatory.

### Process 1

#### NA is an acute synchronized neutrophilic process of mid-sized arteries that is responsible for the saccular aneurysms that could either thrombose or rupture within the first month (Figures 1, 2A, 2B, 2C)

NA starts at the endothelium and sequentially destroys the intima, IEL, media, EEL, and varying amounts of the adventitia of coronary and medium sized non-coronary (i.e., axillary, inter-costal/inter-vertebral, iliac, mesenteric) muscular and elastic arteries. The inflammatory cells are neutrophils and the necrotic wall/tissue is a combination of neutrophils, cellular debris, and fibrin. The remaining adventitia, pericardial, and peri-vascular tissue of the CAA invariably measured less than 1 mm in thickness. In some instances, the aneurysmal vessel consisted of only some pericardial/peri-vascular tissue. Free wall aneurysms were more likely to rupture than those “reinforced” by lateral tissue and/or myocardium. Although necrosis often approached adventitial vessels, focal hemorrhage was rare and appeared to be inconsequential. There was no evidence that NA ever involved small extra- or intra-parenchymal arteries or pulmonary arteries, aorta, or veins.

**Figure 1:**
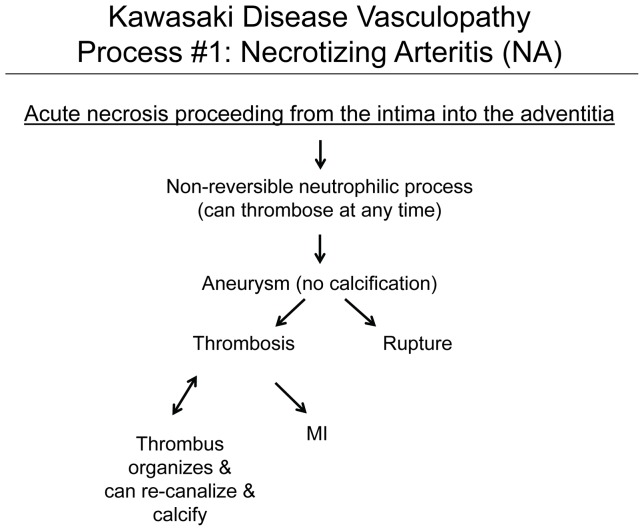
Kawasaki Disease Vasculopathy, Process 1, Necrotizing Arteritis (NA). NA is an acute self-limited, one-time, synchronous process complete within about 2 weeks of fever. It starts at the endothelium of medium sized muscular and elastic arteries and progresses peripherally; involvement of veins, pulmonary arteries, and aorta is not observed.

**Figure 2:**
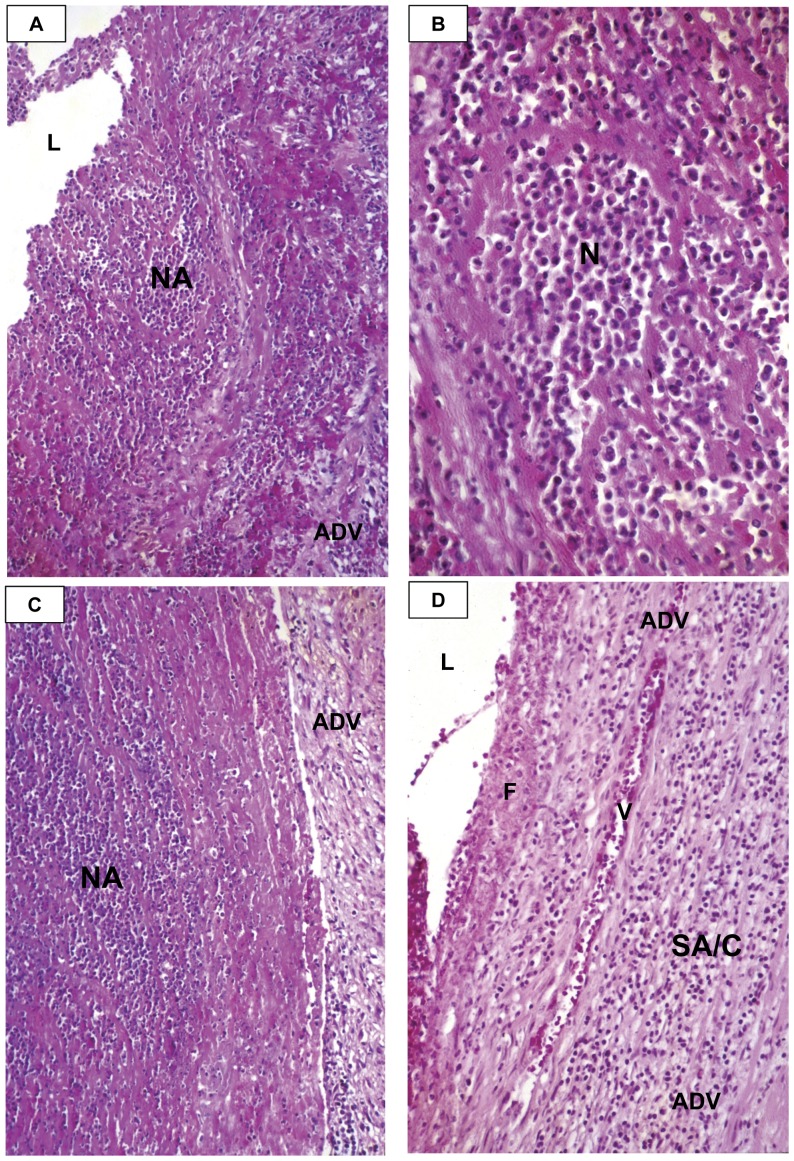
Necrotizing Arteritis (NA) and severe SA/C pan-arteritis. A. Portion of a CA undergoing NA. The friable fragmenting wall is a mixture of neutrophils and debris. The adventitia is virtually obscured by inflammation and RBCs. H&E, case 2, original magnification 16×. B. Higher magnification of an area of CA NA predominantly of neutrophils. H&E, case 2, original magnification 63×. C. The necrotizing process has reached into the adventitia, which is only mildly inflamed. H&E, case 2, original magnification 16×. D. An area of CAA consistent with having undergone severe SA/C pan-arteritis, leaving only adventitia rich in SA/C inflammatory cells, almost exclusively small lymphocytes. Note the longitudinally sectioned vessel and fibrin/RBC lining the luminal surface. H&E, case 11, original magnification 40×. L=lumen, ADV=adventitia, V=vessel, F=fibrin, NA=necrotizing arteritis, N=neutrophils.

NA was a self-limited process, essentially complete within two weeks after onset, a time when specimens are inherently rare. However, many patients who died later had evidence of previous CA and NCA necrosis. Only adventitia remained after NA; the other arterial layers had been sloughed. The residual adventitia could have scattered neutrophils. There was no evidence that NA commenced or recurred after day 14.

The sections from the patient who died on day 13 (Case 2) from a ruptured left anterior descending CAA showed waning NA in the vicinity of the rupture site (Figures 2A, 2B, 2C). He had been diagnosed 24 hours before death, at which time he received IVIG and aspirin. Oblique sections of the ruptured tortuous left anterior descending artery demonstrated the complexity of KD vasculitis/vasculopathy. Closest to the rupture site, there was a mixture of neutrophils and necrotic debris extending from the lumen into the adventitia. Further away, the debris had sloughed and replaced by fresh clot. Other areas displayed active SA/C inflammation and SA/C-LMP lesions. In many KD patient CA, post-NA and active SA/C vasculitis were present simultaneously at different locations along the same and different CA and NCA.

### Process 2

#### SA/C vasculitis is an asynchronous non-neutrophilic inflammatory process predominantly of small lymphocytes and is closely associated with LMP lesions

**Figure 3:**
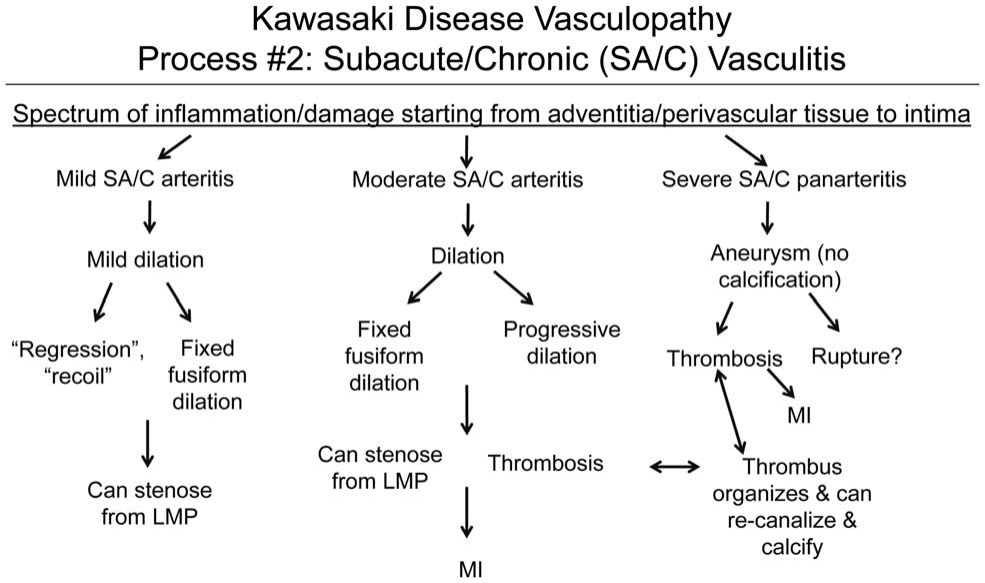
Kawasaki Disease Vasculopathy, Process 2: Subacute/Chronic (SA/C) Vasculitis. A ubiquitous, asynchronous inflammatory process that can begin as early as the first two weeks, and targets medium-sized muscular and elastic arteries. Inflammatory cells: small lymphocytes>>eosinophils & plasma cells>> macrophages. Minimal (subclinical) involvement of veins, pulmonary arteries, & aorta. Triggers Process 3, luminal myofibroblastic proliferation (LMP).

**Figure 4:**
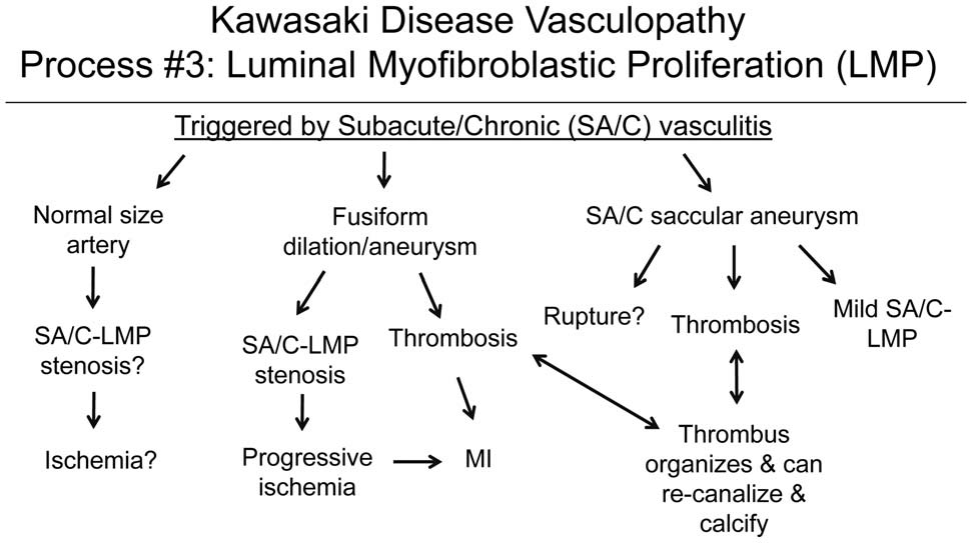
Kawasaki Disease Vasculopathy, Process 3: Luminal Myofibroblastic Proliferation (LMP). A process triggered locally and apparently remotely by Process 2 that can progress to total occlusion. LMP myofibroblasts are ultrastructurally, but not functionally, similar to wound healing myofibroblasts.

**Figure 5:**
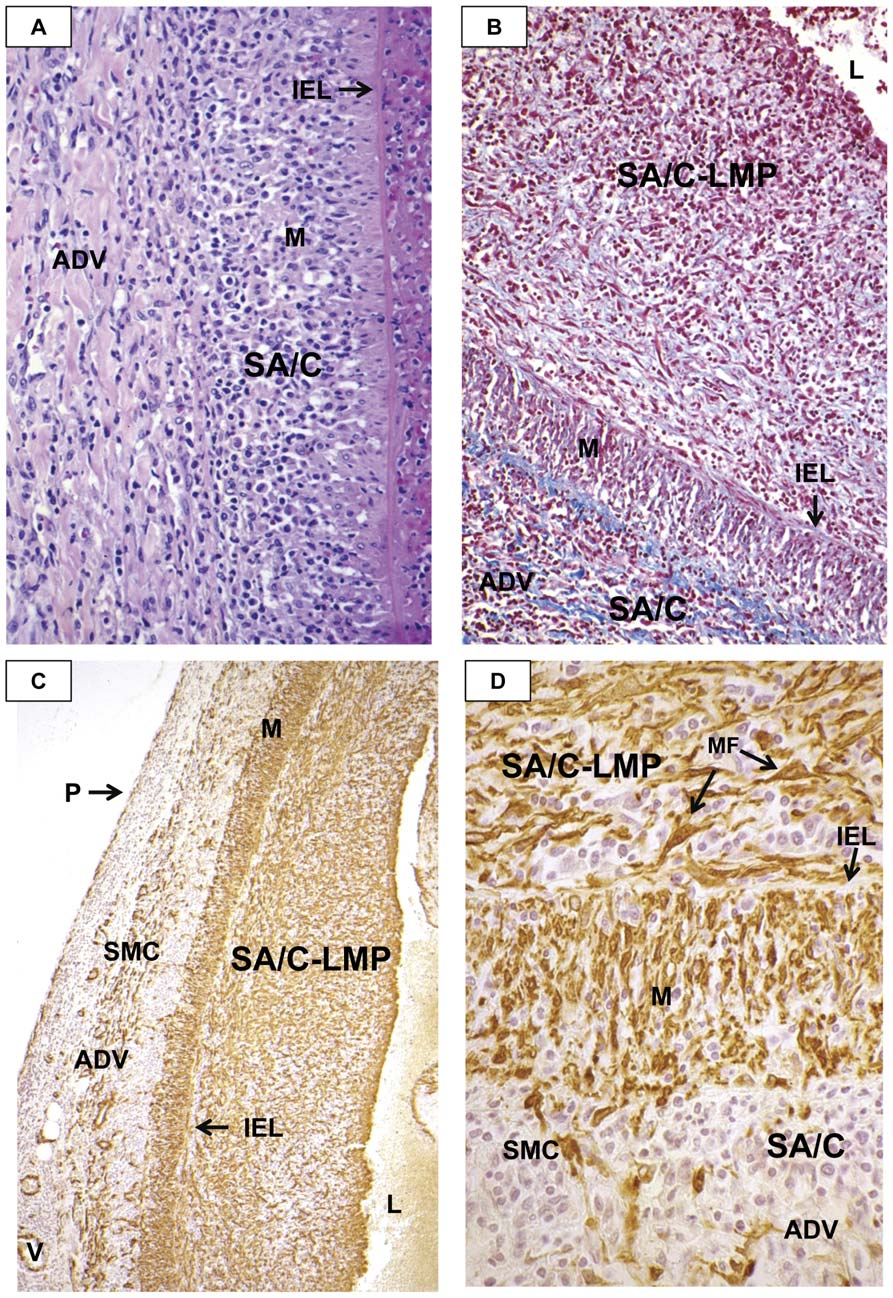
Subacute/Chronic (SA/C) Inflammation in KD CAA. A. SA/C inflammatory cells extending from the adventitia of a CA has almost reached the IEL. The EEL and most of the media are obscured/destroyed by the SA/C. The lumen contains a mixture of RBC, fibrin, and lysing leukocytes, that could indicate the beginning of a clot. H&E, case 11, original magnification 40×. B. The CA adventitia and media are rich in SA/C inflammatory cells. The thick inflammation of SA/C-LMP has obscured most of the myofibroblasts. There is more collagen staining (blue) in the adventitia than protein staining of the ECM of the SA/C-LMP. Note the increase in cellular concentration toward the lumen. Trichrome stain, case 18, original magnification 16×. C. The alpha-SMA stains SMC and vessels in this CA adventitia, SMC in the media, and MF in the SA/C-LMP. Focal areas of IEL remain. Alpha-smooth muscle actin (SMA) immunohistochemistry (IHC), case 18, original magnification 10×. D. At higher magnification, SMCs appear to be entering the media from the adventitia, while large myofibroblasts exit the luminal side into the SA/C-LMP. SA/C inflammation is present in all three layers. Alpha-SMA IHC, case 18, original magnification 63×. M=media, SMC=smooth muscle cells, MF=myofibroblast, ADV=adventitia, IEL=internal elastic lamina, LMP=luminal myofibroblastic proliferation, L=lumen, V=vessel, P=pericardium.

**Figure 6:**
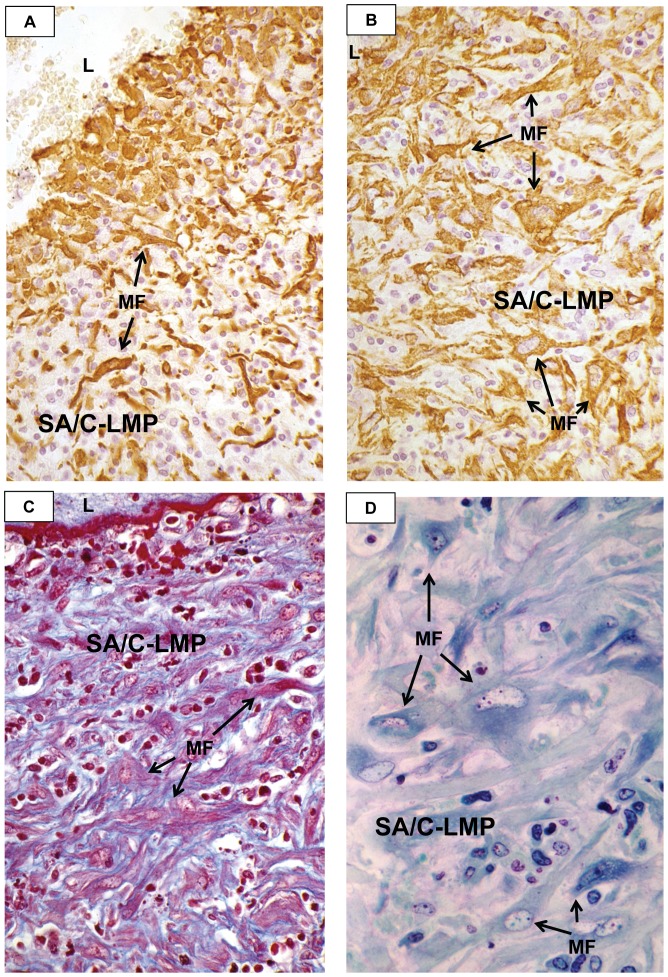
Luminal Myofibroblastic Proliferation (LMP) in KD CAA. A. The peri-luminal portion of the CA SA/C-LMP is rich in polygonal shaped myofibroblasts, while elsewhere the myofibroblasts are more pleomorphic. The background is mostly small lymphocytes. Alpha-SMA IHC, case 18, original magnification 63×. B. At higher magnification, the myofibroblasts in this area of SA/C-LMP resemble a culture of pleomorphic mesenchymal cells. The SA/C background is especially rich in small lymphocytes. Alpha-SMA IHC, case 13, original magnification 100×. C. The LMP resembles a “syncytium” of pleomorphic MF with red-staining actin and blue-staining pro-collagen. SA/C nuclei stain dark red. There is some blue staining of the ECM. The luminal lining (top) consists of RBCs and dark red staining fibrin. Trichrome stain, case 13, original magnification 100×. D. The SA/C-LMP contains large, pleomorphic myofibroblasts with large nuclei and several small nucleoli. The ECM is difficult to delineate. Trichrome stained plastic section, case 13, original magnification 160×. L=lumen, MF=myofibroblast, LMP=luminal myofibroblastic proliferation.

**Figure 7:**
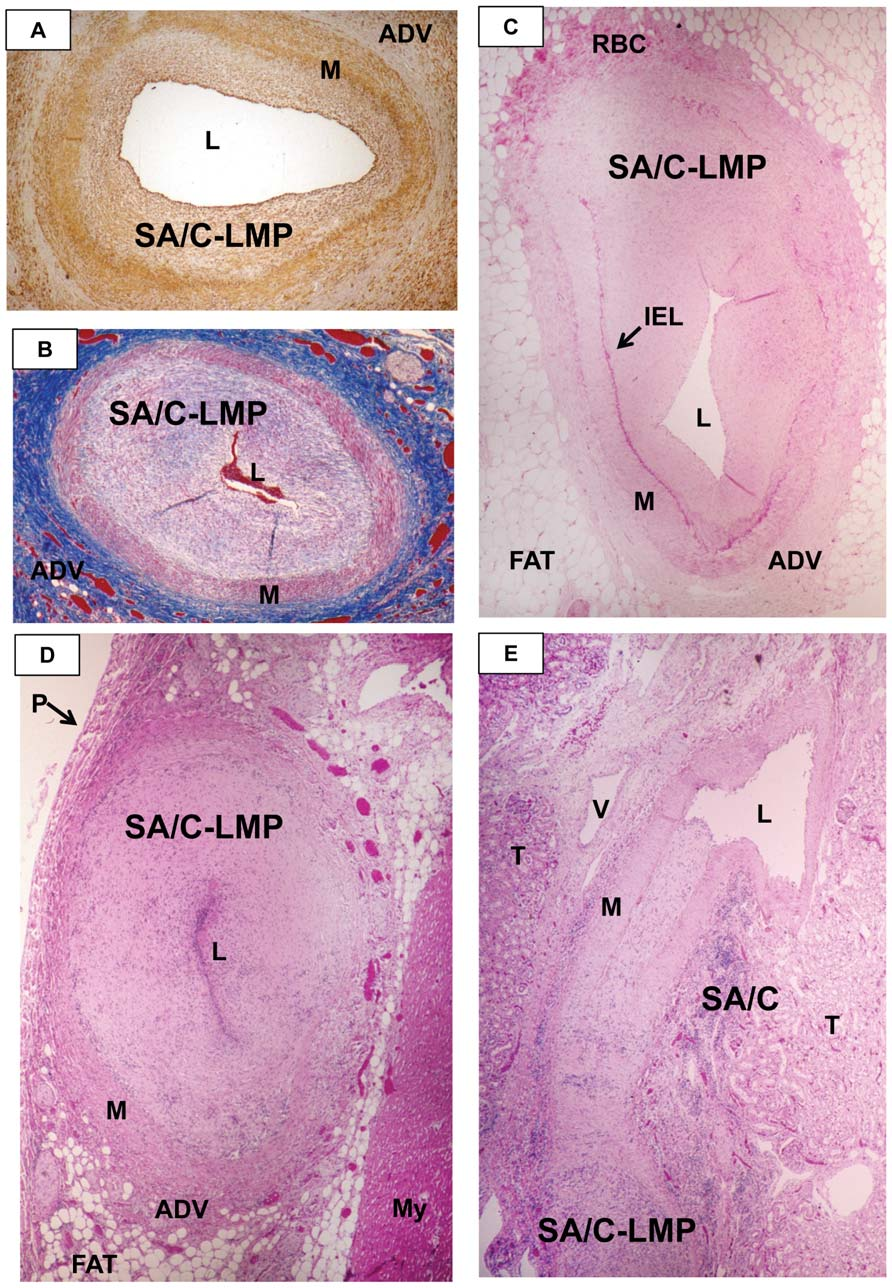
SA/C-Luminal Myofibroblastic Proliferation (LMP) causes narrowing of CA in KD patients. A. A tear drop-shaped CA with SA/C-LMP is about 50% narrowed. The actin-rich myofibroblasts and medial and adventitial SMCs stain brown. The residual media is variably thinned. Alpha-SMA IHC, case 11, original magnification 10×. B. Oval-shaped SA/C-LMP CA is virtually occluded. The MF and medial SMC stain red and there is a relatively little ECM (blue) in the LMP. Except for some thinning, the media is almost completely intact. The adventitia is densely collagenized. Trichrome stain, case 28, original magnification 10×. C. The SA/C-LMP in this elongated CA is eccentric having started at the end (top) where the media and IEL had been destroyed and progressed toward the end, which still contains media and IEL (bottom). H&E, case 26, original magnification 10×. D. An oval CA is occluded by SA/C-LMP, of relatively low SA/C cellularity concentrated around the lumen. There are several small areas of minimally preserved media. No media is present on the side with the hyper-vascular adventitia, where the process likely began. H&E, case 27, original magnification 10×. E. The SA/C-LMP progressed from the inflamed/damaged end (bottom) of this tangentially-sectioned renal artery almost reaching the opposite end (top), where the lumen is visible and there is intact media and IEL are intact. H&E, case 27, original magnification 10×. L=lumen, M=media, ADV=adventitia, LMP=luminal myofibroblastic proliferation, RBC=red blood cells, IEL=internal elastic lamina, V=vein, I=inflammation, T=renal tubules, P=pericardium, My=myocardium.

**Figure 8:**
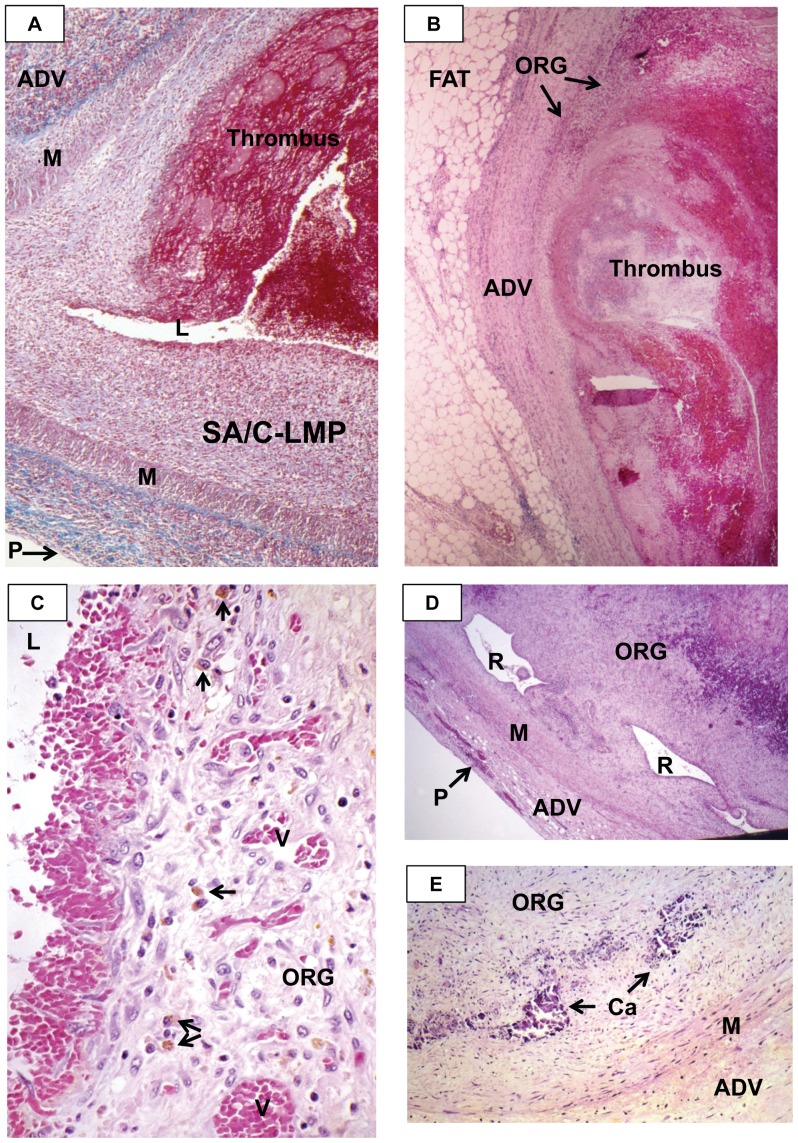
Thrombi in coronary artery aneurysms (CAA) of KD patients. A. A CA already severely compromised by SA/C-LMP is virtually occluded by a superimposed fresh thrombus (dark red) that blends into the SA/C-LMP. Trichrome stain, case 18, original magnification 16×. B. Part of a NA CAA occluded by a fresh thrombus. There is a small area of organizing thrombus. Since only mildly inflamed fibrotic adventitia remains, it is not possible in this section to distinguish between NA and SA/C as the etiology. H&E, case 11, original magnification 10×. C. A small portion of CAA thrombus. The vascular granulation tissue has a loose matrix containing few free RBC, spindle cells and mononuclear cells, a few of which are macrophages containing brown hemosiderin blood pigment. Spindle cells are reaching into the luminal RBCs. H&E, case 22, original magnification 63×. D. The oldest peripheral thrombus in this CAA is re-canalizing. There are superimposed fresher thrombi. Since the CAA still has some remaining media, the aneurysm likely resulted from severe SA/C pan-arteritis. H&E, case 37, original magnification 10×. E. This organized SA/C CAA thrombus has peripheral clumps of calcium. Some of the media is still visible (upper right). H&E, case 19, original magnification 16×. L=lumen, LMP=luminal myofibroblastic proliferation, M=media, ADV=adventitia, ORG=organizing thrombi, R=recanalized, Ca=calcium, V=vessel.

**Figure 9:**
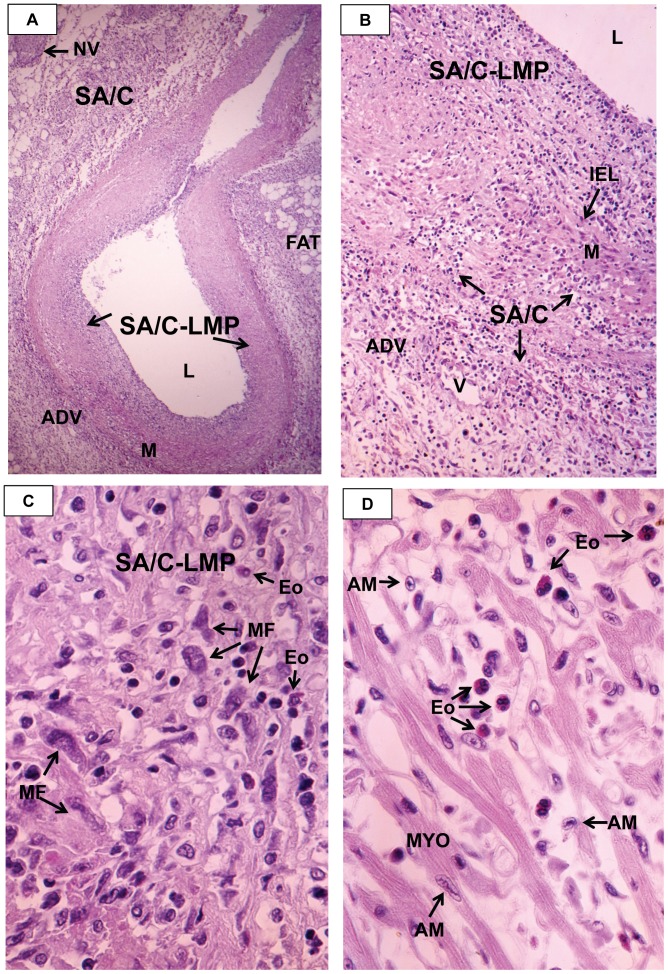
Myocarditis. A. Low magnification H&E of a poorly-preserved hourglass-shaped epicardial CA showing varying degrees of SA/C peri-arteritis, transmural SA/C, and minimal preserved media. H&E, case 1, original magnification 10×. B. Perivascular and transmural SA/C inflamed adventitia and heavily damaged media with some discernible SMC and IEL, and SA/C-LMP with scattered pleomorphic myofibroblasts. There are some eosinophils intermixed with the small lymphocytes. H&E, case 1, original magnification 16×. C. A higher magnification of a typical area of SA/C-LMP with prominent amphophilic myofibroblasts in a background of mostly small lymphocytes, scattered eosinophils, and likely macrophages in a fibrillar ECM that shows some artifactual spaces. H&E, case 1, original magnification 25×. D. A typical area of the highly edematous interstitial myocarditis especially rich in eosinophils, with scattered lymphocytes, macrophages, and plasma cells. Note the longitudinally-sectioned caterpillar-shaped and cross-sectioned, owl eye-shaped (unidentified) Anitschkow chromatin pattern in a myocyte and two unidentified cell nuclei, respectively. H&E, case 1, original magnification 40×. L=lumen, NV=nerve, IEL=internal elastic lamina, Eo=eosinophil, AM=Anitschkow myocyte, MF=myofibroblast, V=vein, ADV=adventitia, M=media.

It can begin in the first 2 weeks but is also observed for months to years after diagnosis (Figures 2D, 3, 4, 5, 6, 7, 8, 9). SA/C vasculitis and its associated LMP (SA/C-LMP) process were observed in the coronary arteries of all 41 cases, even at day 10 (Case 1, Figure 9B). In cases where more vascular tissue was available, a greater extent of CA and NCA involvement was documented. Small epicardial CA could have from no to severe SA/C vasculitis and SA/C-LMP. Intra-myocardial vessel SA/C inflammation was minimal and random. There did not appear to be any mid-sized muscular or elastic artery that escaped SA/C and SA/C-LMP, with the exception of the central nervous system, which never displayed evidence of any of the three processes. Some cases had relatively mild cerebrovascular infiltrates of predominantly lymphocytes not associated with parenchymal or vascular pathology. The most frequently involved non-coronary arteries were renal, mesenteric, axillary, iliac, peri-pancreatic, and hepatic. There were infarcts of the kidneys, adrenals, and spleen that were apparently asymptomatic at the time of death. Although SA/C and SA/C-LMP frequently involves veins (epicardial or otherwise), pulmonary arteries, and the aorta, the degree of involvement was relatively minimal, there were no associated thrombi or apparent clinical significance. Two autopsy reports described multiple sub-renal aortic aneurysms of less than one centimeter in diameter; unfortunately no blocks/sections or photos were available from these aneurysms.

The inflammatory cells of SA/C were predominantly small lymphocytes, with varying numbers of plasma cells (often in clusters, but rarely containing Russell bodies) and eosinophils (including band forms), scattered macrophages, and rare neutrophils. “Atypical” activated mononuclear cells were rarely observed. Granulomas and granulomatous inflammation are neither features of KD vasculitis nor non-vascular lesions. The only CD68-positive macrophages observed were apparently in the process of scavenging medial and adventitial debris resulting from SA/C vasculitis. SA/C inflammation commenced in the peri-vascular/adventitial vaso vasorum and progressed luminally (Figure 5A), in the opposite direction compared with NA.

The degree of SA/C inflammation and accompanying tissue damage formed a broad spectrum ranging from mild to severely destructive pan-arteritis/peri-arteritis, which could lead to saccular CAA and thrombosis. The initial site of vessel inflammation was frequently quite obvious and appeared to progress circumferentially accompanied by the induced luminal SA/C-LMP, until the intra-luminal lesion was of relatively equal thickness. When the inflammation/damage was relatively mild, the media was virtually intact and IEL and EEL were without evidence of dilation or distortion. When the degree of inflammation/damage was somewhat greater, the artery apparently could initially dilate and then return to its normal morphology/diameter by “recoiling” or it could remain fixed in a mildly dilated fusiform state, both with substantial amounts of preserved media and elastic; their SA/C lesions could progress. At the next level of severity/intensity, the inflamed/damaged arteries could progressively dilate into a saccular aneurysm with relatively little remaining IEL, EEL, and media, and could thrombose. Occasionally, small amounts of remaining IEL calcified, which enhanced their visualization in H&E sections.

Arteries that experienced the most severe SA/C arteritis underwent a form of necrosis whereby the vascular wall resembled layers of a peeling onion, but without neutrophils. In contrast to saccular CAA resulting from NA, after SA/C there were often some foci of preserved media and IEL. The remaining adventitia contained varying amounts of SA/C inflammation. The resulting saccular CAA frequently thrombosed but apparently did not rupture. Thus, while fusiform CAA are due to SA/C inflammation, saccular CAA can be due to either NA or severe SA/C. More saccular CA and NCA aneurysms appeared to be due to severe SA/C than NA. Some saccular aneurysms developed relatively minor LMP lesions with little luminal narrowing, apparently because destruction of the media limited the available SMC for transition to myofibroblasts. While the first saccular CAA thrombosis caused by either NA or SA/C could be fatal, alternatively it could start organizing, and there could be additional thrombotic episodes, any one of which could be fatal. The commencement of SA/C saccular aneurysm formation occurred in the first 1–2 months, while thrombosis could occur at any time.

The child (Case 13) who died from a ruptured iliac artery aneurysm between 4 and 5 weeks had evidence of both severe SA/C inflammation/damage and post-NA changes, with sloughing of necrotic debris. Iliac artery sections also showed varying degrees of stenosing SA/C-LMP lesions.

Another child (Case 11) who died from a myocardial infarction three to four weeks after presentation had a single CA plus several NCA that were in the process of extreme SA/C arteritis, accompanied by sloughing and aneurysm formation. An axillary artery aneurysm contained a friable thrombus with no evidence of organization. Two inter-costal/inter-vertebral arteries were in the process of shedding their media and part of their adventitia and were already quite dilated. One CAA section showed post-NA features.

### Process 3

#### LMP is a progressive asynchronous intra-luminal stenosing process of SMC-derived myofibroblasts of the type described in wound healing by Gabbiani [28] plus their matrix products and SA/C inflammation (SA/C-LMP)

It is neither granulation tissue nor does it show organization, “remodeling”, or recanalization (Figures 4, 5, 6, 7, 8, 9, 10, 11, 12, 13, 14).

**Figure 10:**
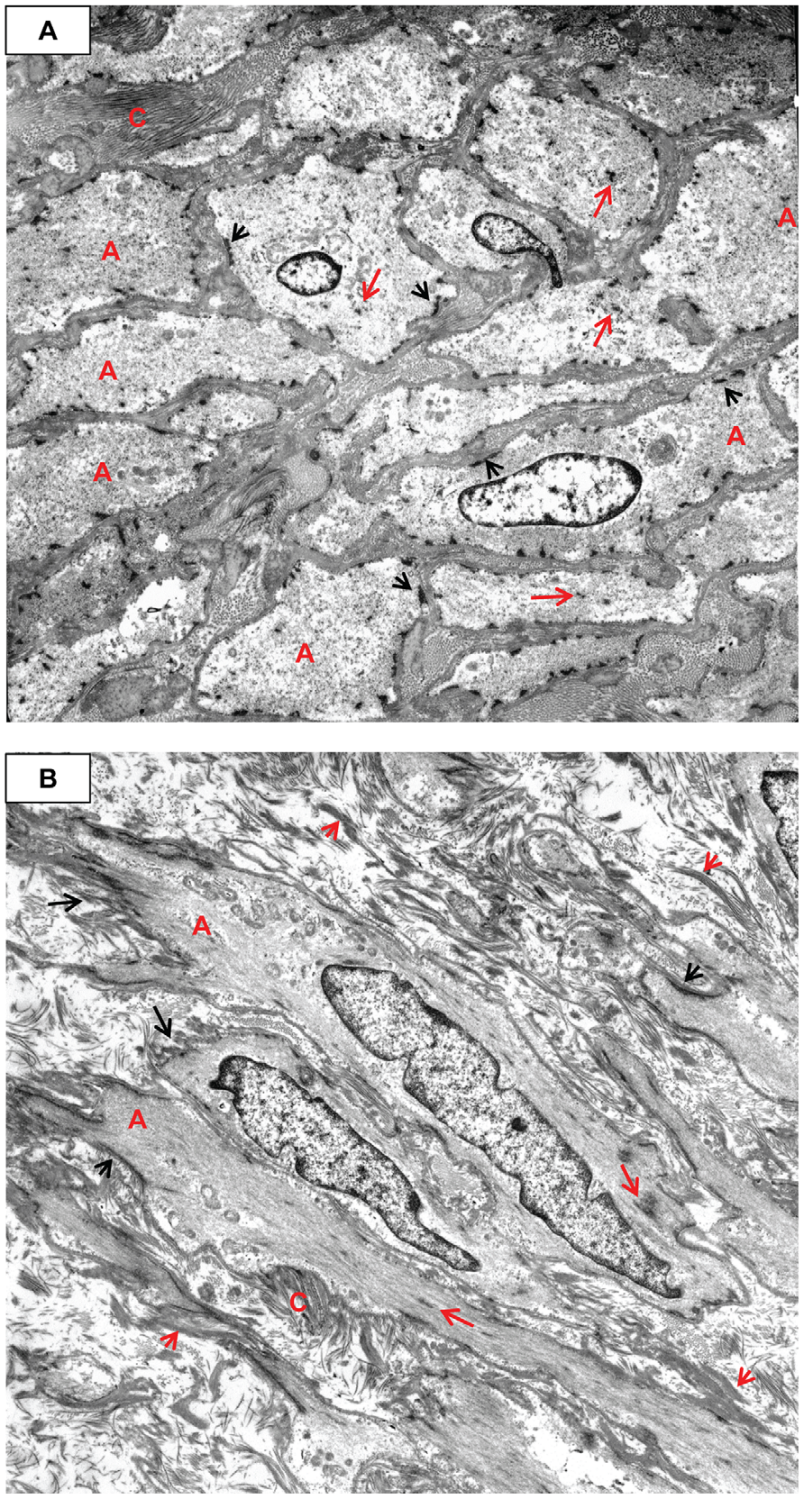
Early transitional changes of medial SMC into myofibroblasts. **A**) Tangential section of media showing variably oriented electron dense banded collagen (e.g., C). The SMC are somewhat swollen, yet the actin (A)/dense bodies (long red arrows) are still apparent. The external lamina is obscured by the ECM. The dense plaques (short black arrows) are visible. Case 32, original magnification 5,000×. **B**) Longitudinal section of medial SMCs commencing their transition to MF. There is abundant actin (A) with dense bodies (long red arrows), dense plaques (short black arrows), shedding external lamina (short red arrows), and serrated nuclei. Electron dense banded collagen production has increased, and there is fibronectin (long black arrows). Higher magnification showed abundant pinocytic vesicles. Case 32, original magnification 4,000×.

**Figure 11:**
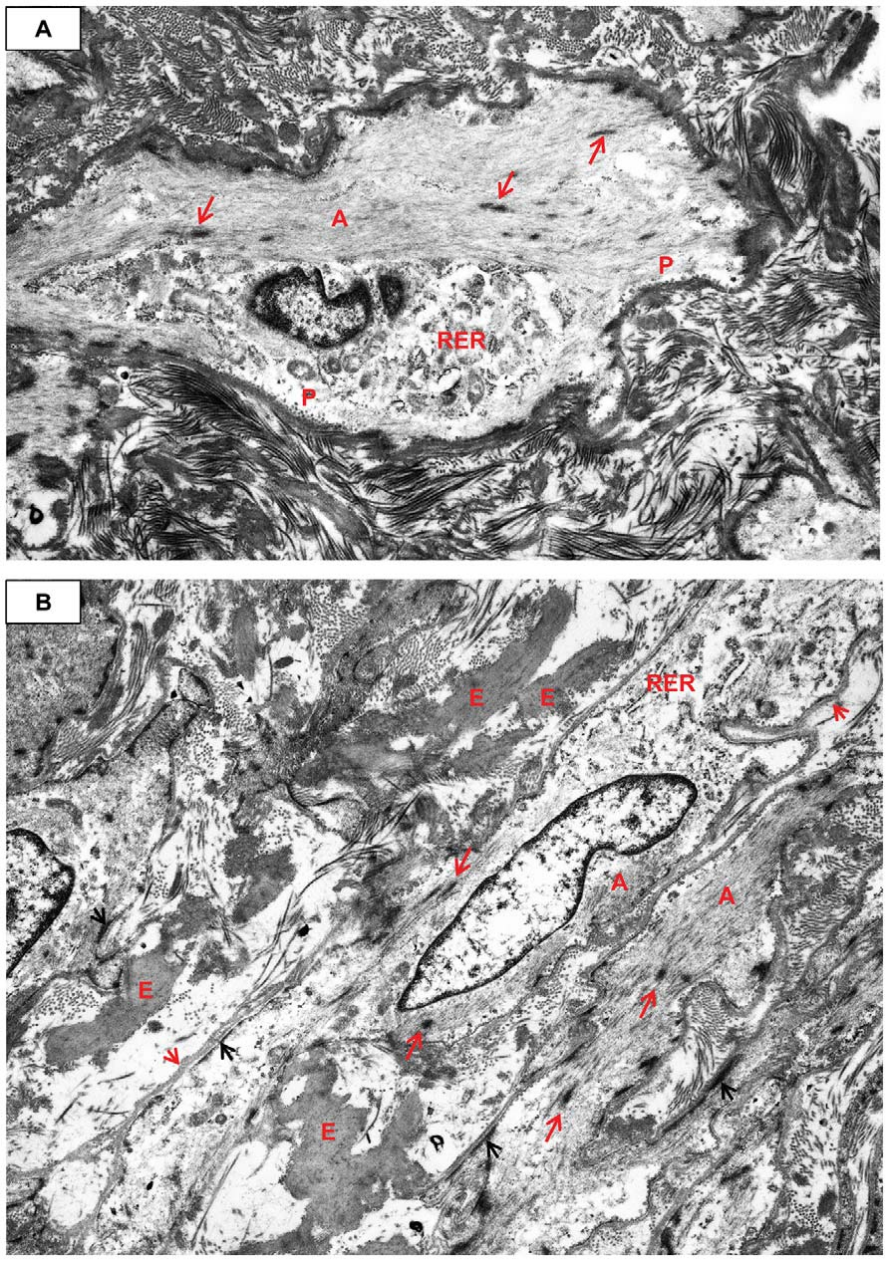
Increase in myofibroblast RER and pleomorphism, as well as intercellular elastin. **A**) The actin (A)/dense bodies (long red arrows) share the cytoplasm with RER. The extracellular matrix is composed almost exclusively of haphazardly arranged electron dense banded collagen. The ECM obscures the dense plaques and external lamina. Pinocytic vesicles (P) are visible. Case 32, original magnification 5,000×. **B**) Dense plaques (short black arrows), actin (A), and dense bodies (long red arrows) have decreased and there is more RER. Note the abundant electron dense intercellular elastin (E), mixed with the collagen. Note the external lamina (long black arrows). Case 32, original magnification 5,000×.

**Figure 12:**
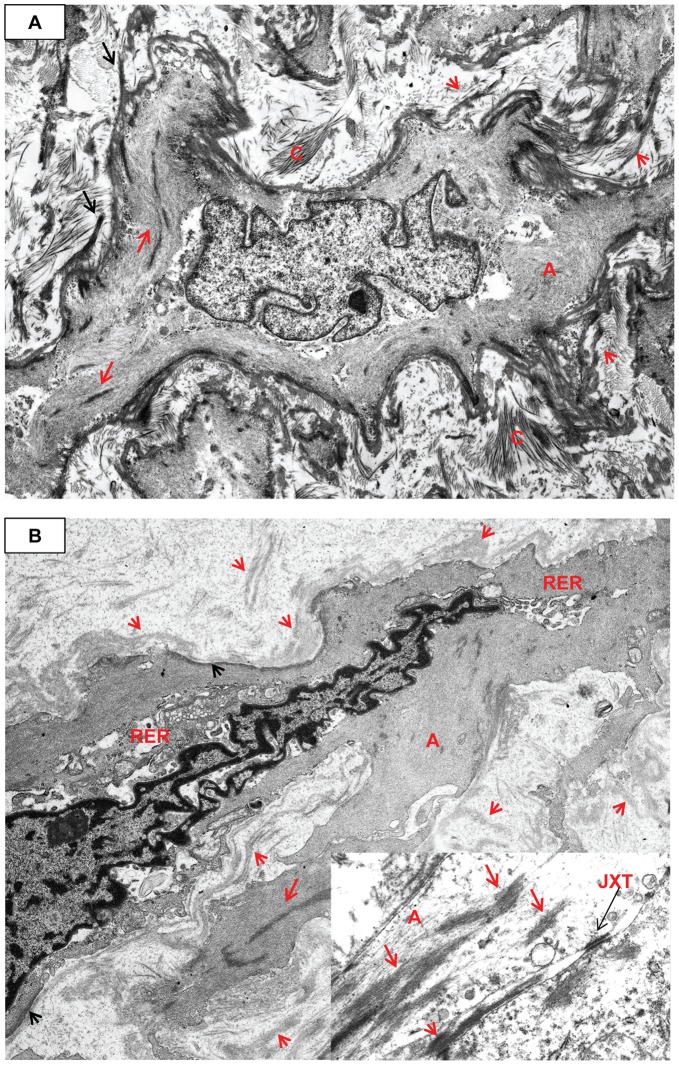
Myofibroblast fibronectin, shed external lamina, and intercellular junction. **A**) There is copious actin (A) with prominent dense bodies (long red arrows) in this large pleomorphic myofibroblast. The nucleus is also large and irregular. Note the electron dense fibronectin (long black arrows). The ECM is loose and contains shed external lamina (short red arrows). Case 32, original magnification 5,000×. **B**) The cell has two prominent visible cytoplasmic extensions and is shedding strips of external lamina (short red arrows) into a loose stroma of granular-fibrillar ECM, with no banded collagen. Dense bodies (long red arrow), actin (A) and RER are present within the cell. Case 26, original magnification 5,000×. Inset: A non-specific junction (JXT) joins two MF. Actin (A) is present with very prominent dense bodies (long red arrows). Case 13, original magnification 10,000×.

**Figure 13:**
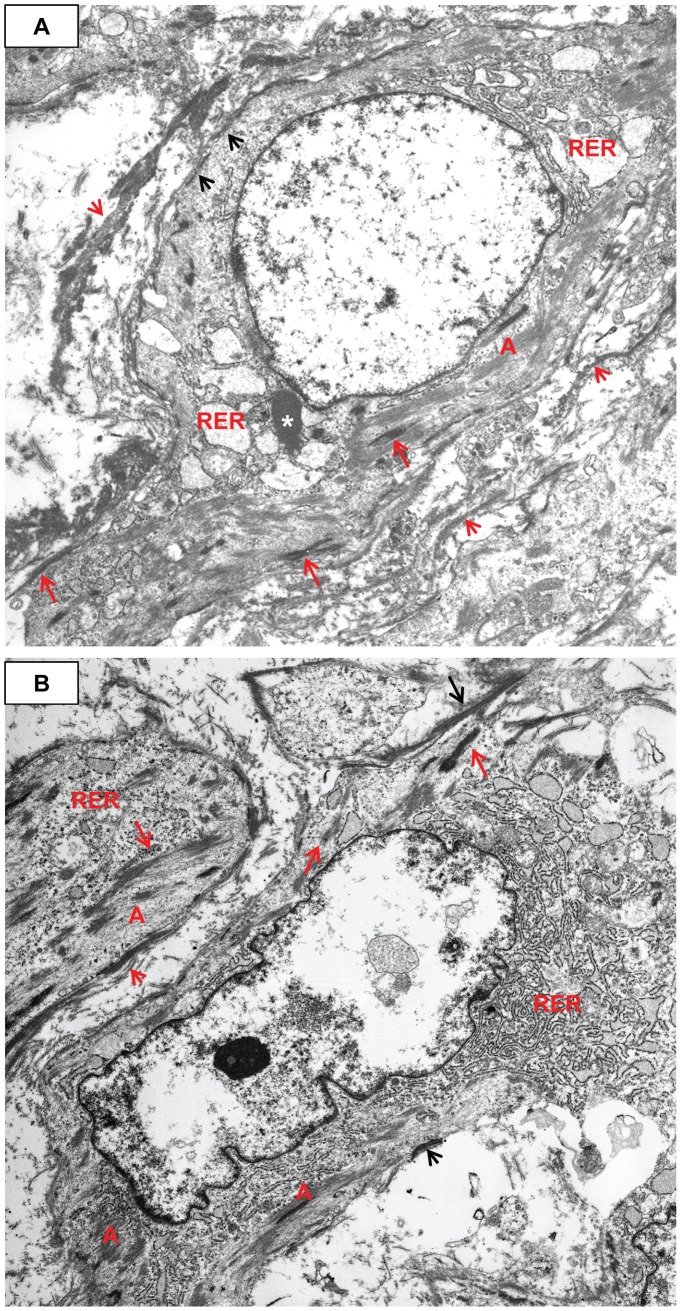
Copious cytoplasmic RER and shed external lamina and fibronectin. **A**) The slightly tangentially sectioned cell has about equal RER and actin (A) with dense bodies (long red arrows). There is a single large primary lysosome (*), shed external lamina (short red arrows), and relatively sparse dense plaques (short black arrows). Case 13, original magnification 5,000×. **B**) A MF with more RER than peripheral actin (A)/dense bodies (long red arrows). The nucleus is large and irregular with a prominent nucleolus and apparently lost some chromatin during processing. Shed external lamina (short red arrow) and fibronectin (long black arrow) are prominent. Case 13, original magnification 4,000×.

**Figure 14:**
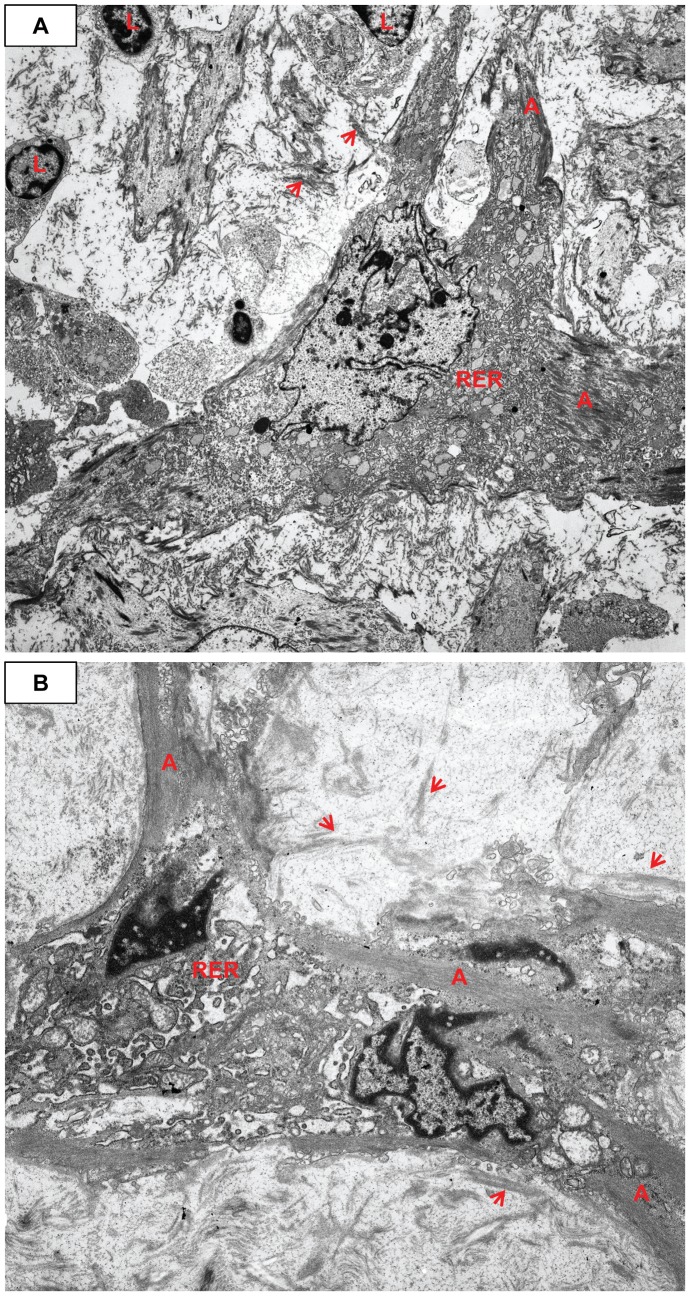
Pleomorphic mono- and binucleated myofibroblasts, abundant RER and SA/C lymphocytes. **A**) RER dominates the cytoplasm of this stellate myofibroblast with a complex nucleus and at least two small nucleoli; some actin (A) is present. The matrix contains some shed external lamina (short red arrows) and fine filamentous collagen. There are typical small lymphocytes (L) with high nuclear to cytoplasmic ratios. Case 13, original magnification 1,700×. **B**) This MF is bi-nucleated and has very dilated, complicated profiles of RER plus actin (A). The loose ECM is dominated by shed external lamina (short red arrows). Case 26, original magnification 5,000×.

The lesions were typically circumferential and relatively symmetric, ultimately leaving a slit-like lumen (Figure 7). Longitudinal and tangential sections could display a variably attached obstructing SA/C-LMP lesion extending free within the lumen. Determining the full extent and degree of the luminal lesions requires examining multiple sequential cross sections (“bread-loafing”). SA/C was documented in over 20 different arteries in our cases, varying somewhat from patient to patient.

Historically, the luminal lesions of KD have been considered to arise from the intima/sub-intima; however, this area is typically denuded by the SA/C inflammatory process. LMP lesions directly abut either the IEL or the media, depending on the status of the IEL. Therefore, considering its intraluminal location, myofibroblast content, and proliferative nature, it seems more appropriate to use the term “luminal myofibroblastic proliferation”. Based on the intimate association, the SA/C inflammatory process appears to be key to LMP development.

Interestingly, it was not unusual to see arterial sections with mild to totally occlusive LMP or media pathology that displayed little to no apparent SA/C inflammation. This could indicate that the inflammation was out of the plane of section, had resolved over time without leaving media damage, or that active SA/C inflammation in a distant part of the same or another vessel was involved in producing circulating factor(s) that could cause development of LMP at *remote* locations. LMP lesions could be associated with thrombosis.

All transplanted hearts had CAA, myocardial infarctions of varying vintages, and SA/C-LMP lesions. A few had fresh CA thrombi, while one had a ruptured CAA.

SA/C-LMP was present in the asymptomatic CAA resected 16 years after acute KD (Case 34). Since the available sections showed some surviving media and IEL, the CAA had likely been caused by SA/C pan-arteritis and not NA.

SA/C-LMP lesions most resemble what was described in KD pathology studies from the 1970s as stages 4 and 5 [19] or stage III [20], and are consistent with the progressive luminal narrowing that has been referred to variably as intimal hyperplasia, fibro-cellular thickening of the intima, intimal fibrosis or proliferation, granulation, obliterative panarteritis, and smooth muscle or fibro-intimal hyperplasia, hypertrophy, or thickening. These terms are based on H&E and occasional histochemical stains, without LM of semi-thin resin sections, TEM or IHC.

### The SMC-derived Myofibroblast of KD LMP Behaves “Pathologically”

The pathognomic cell of LMP is neither a fibroblast nor a SMC, although relatively small numbers of either or both of these cells can also be present. Even though it is SMC-derived, the LMP myofibroblast has the classic ultrastructural features of the pathognomonic fibroblast-derived wound healing myofibroblast described by Gabbiani [28]. The stimulus for LMP and wound healing myofibroblasts may be similar. However, the LMP myofibroblast remains activated, causing progressive luminal narrowing to the point of total obstruction without undergoing apoptosis, in contrast to the life-saving terminally differentiated myofibroblast of wound healing.

While still situated within the media, SA/C-stimulated SMC enlarge, become increasingly pleomorphic, and produce more banded collagen and elastin fibers than usual (Figures 10, 11). These transitioning cells tend to re-orient from circumferential to perpendicular to the media as they migrate luminally either thru pores or gaps in the IEL, and along with SA/C inflammatory cells, emerge from the luminal aspect of the media to create enlarging SA/C-LMP lesions.

Extrapolating from the ultrastructure, the SMC to myofibroblast transition can be readily demonstrated by light microscopy in H&E and trichrome-stained sections and by SMA IHC. Adventitial SMC appear capable of entering the peripheral media, as if in the act of media re-population. Where there are breaks in the media/IEL, adventitial SMC appear capable of migrating all the way into SA/C-LMP lesions as they transition into myofibroblasts (Figure 5D).

The largest, most pleomorphic myofibroblasts tend to be concentrated near the lumen. However, they can be near the media or even distributed throughout the entire SA/C-LMP lesion, giving it the appearance of a culture of pleomorphic mesenchymal cells. Mitotic myofibroblasts were occasionally observed, most often in the vicinity of the luminal surface. The trichrome stain can demonstrate the dual nature of myofibroblasts; the peripheral actin stains red and the central pro-collagen- containing RER stains blue (Figures 6C, 6D, 7B).

Occasional stretches of a new, “second” IEL can be observed under the “endothelial” cells lining the luminal surface in areas of a section where there was also normally located IEL. It is not clear whether this indicates that the LMP lesion had ceased expanding and was developing a new intimal lining.

Prolonged post-mortem or surgical hypoxia typically creates artifacts such as cytoplasmic edema/swelling (hydropic change), interstitial edema, and sloughing of the endothelium. While in some specimens “normal” vessels had some remaining endothelial cells, LMP lesions were frequently lined by one or more layers of pleomorphic, hyperchromatic, amphophilic H&E staining cells that were strongly positive for both Factor VIII-related antigen and SMA (Figure 6A).

Ultrastructurally, the transition of SMC into myofibroblasts is characterized by a progressive increase in central dilated profiles of RER and a proportional decrease in bundles of actin with dense bodies (“stress fibers”) [28] (Figures 10, 11, 12, 13, 14). TEM is the only definitive means to identify myofibroblasts, since there is no specific IHC marker. As with wound healing myofibroblasts, the stress fibers of LMP myofibroblasts are associated with sub-plasmalemmal dense plaques and electron dense, extracellular, fibornectin filaments forming “fibronexus” complexes (Figures 12A, 13B). However, indicative of their SMC origin, LMP myofibroblasts can maintain varying amounts of external lamina material and pinocytic vesicles (Figure 10B, 11A). Mesenchymal or adherens- type junctions are seen, but gap junctions have yet to be detected. The myofibroblast nuclei vary in size and shape and contain one or two prominent nucleoli. They are often in intimate contact as if in the act of fusion, while occasional bi-nucleated cells are identified (Figure 14B).

### The ECM of LMP Lesions

The ECM of the LMP lesions varied in proportion to the cellularity and staining density as seen by the H&E (pink) and trichrome (blue) stains (Figures 5,6). Ultrastructurally, it consisted of variably-oriented and electron dense banded collagens and loose non-banded collagen fibrils. Parallel rows of banded collagen fibrils oriented perpendicular to and intimately associated with the plasma membrane of myofibroblasts were observed, typical of cells actively producing collagen that polymerizes at the plasma membrane. Tight arrays of electron dense fibronectin filaments both extended from the plasma membranes and shed into the stroma. Lighter staining, finely filamentous/flocculent external lamina collagen was seen both attached to the plasma membrane and shed into the stroma. The ECM also contained varying amounts of glycosaminoglycan particles and elastin fibers (Figures 10, 11, 12, 13, 14).

### Distinguishing SA/C-LMP Lesions from Organizing and/or Organized Thrombi

Thrombi were seen intercalated into the damaged adventitia of CAA, SA/C inflamed/damaged vessel walls, or overlying/blending into the luminal surface of SA/C-LMP lesions (Figure 8). Organization began in the oldest peripheral thrombi (closest to the remaining vessel wall), with mitotically-active endothelial cells of budding capillaries migrating from the adventitia into the clot. Organizing thrombi could undergo progressive collagenization and re-canalization. In all but one case, the re-canalized vessels were typically elongated, parallel to the vessel wall and aligned with the flow of blood, and were lined by endothelium (Figure 8D). In case 33, a single organized thrombus contained a complex of several small equal-sized “mature” arteries with intima, media, and adventitia. However, there was also focal SA/C inflammation and SA/C-LMP, which was not present in published pictures of similar lesions [29], [30], [31]. As opposed to the classic form of organized thrombus, these angiomatoid neo-vessels did not contain blood, and differ from what is observed in atherosclerosis, organized pulmonary emboli, and pulmonary hypertension (e.g., plexiform pattern) [32], [33]. The reasons for the two forms of re-canalization are not clear.

Neither SA/C-LMP nor LMP lesions underwent any form of re-canalization, but could show perpendicularly-oriented, arteriole-size feeder vessels extending from the adventitia through intact media or media gaps, occasionally reaching the lumen.

A feature virtually exclusive to late organizing or organized thrombi were aggregates of coarse calcium, often associated with the re-canalization (Figure 8E). Only a small deposit of calcium was seen in a single LMP section from one case. Neither medial nor adventitial calcification was noted in KD, including the remaining adventitia of CA and NCA aneurysms.

During organization of thrombi, there is an increase in both free and intra-macrophage golden-brown hemosiderin pigment derived from RBCs that had leaked from the budding capillary bed (Figure 8C). Hemosiderin pigment appeared capable of persisting indefinitely in organized thrombi.

### Myocarditis

“Myocarditis” was listed as the cause of the earliest death in our series (Case 1, day 10). However, unlike the few previous descriptions of KD myocarditis, there was no myocyte degeneration or necrosis nor damaged intra-mural vessels. The two most striking myocardial features of this unique KD case were the abundance of mature eosinophils and the marked intercellular and perivascular edema (Figure 9D). Lymphocytes and some plasma cells and macrophages were also seen, but only rare neutrophils. The only cellular pathology observed in the several available sections from the same CA/myocardium block was reversible foci of swollen (hydropic) sub-endocardial myocytes with few contraction bands. The pronounced edema of this “eosinophilic-type” myocarditis would presumably have created a “flabby”, poorly contractile heart and likely a dysfunctional conduction system, leading to acute heart failure. In addition to the “eosinophilic-type” myocarditis, the section contained a figure-eight shaped CA showed SA/C pan-arteritis, SA/C-LMP, pericarditis (especially near the inflamed CA), and several mildly inflamed epicardial nerves (Figures 9A, B, C). The few small epicardial arteries and veins were histologically normal. The endocardium was thickened and showed mild SA/C inflammation and edema.

Two cases, a transplant (case 33) and an autopsy (case 19) had extensive myocardial infarctions plus an atypical myocyte process that resembled myocarditis. The involved myocytes and their nuclei varied markedly in size and shape. Multiple nuclei could be crowded together, nucleoli were typically multiple, and chromatin had a “salt-and-pepper” pattern. Mitotic figures were present, some of which were atypical, even “bizarre” and “anaplastic-looking”. These unusual myocytes were not hypertrophied cells with “box-car”-shaped nuclei, which are frequently observed around older myocardial infarcts. Their etiology is under investigation.

Several other patients had a “pseudo-myocarditis”, where SA/C inflammatory cells spread into myocardium from contiguous inflamed epicardial CA. There was no indication that the infiltrated myocardium was damaged. Thus, depending on the plane of section and/or whether the block contained CA, the myocardium could appear inflamed (“pseudo-myocarditis”).

Multinucleated giant cells were not a feature of CA or NCA vasculitis or vasculopathy in any of our cases [20], [34].

### Pericarditis and Endocarditis

All cases had some degree of pericarditis and endocarditis. The pericardial inflammation was most intense in the vicinity of or overlying CA undergoing SA/C pan/peri-arteritis. Foci of granulation tissue and fibrosis and a covering of fibrinous exudate were occasionally present.

SA/C-induced endocarditis varied in extent, lesional thickness, fibrosis, elastosis, and inflammatory intensity. The endocarditis could directly extend to involve valves, even resulting in their necrosis. However, the endocarditis did not extend into the adjacent myocardium, which frequently showed reversible hydropic degeneration and contraction bands, and even irreversible coagulation necrosis. The degree of fibrosis (dense bands) and density and widening of elastic fibers increased approaching the myocardium. The endocardial lesions occasionally had a longitudinal row of SMC.

### Aortitis

The aortic findings were surprising, given the larger size of the aorta, its prominent elastic laminae, and the apparent absence of significant pathology of other large vessels such as the pulmonary arteries. It is unfortunate that the aortic “aneurysms” described in one autopsy report were not embedded. Though aortas could appear grossly normal random sections often revealed histopathology. There could be mild to moderate lymphocytic inflammation of the vaso vasorum and of the wall with irregular gaps. The intima varied from totally normal to edematous and inflamed. Although most of the inflammatory cells were lymphocytes, some cases had sections of aorta with focal and/or diffuse infiltrates of CD68-positive macrophages, sometimes multinucleated, both foreign body and Langhans types. However, features of atherosclerosis, such as lipid-laden macrophages or SMCs (foam cells), calcium, and cholesterol were not present.

### Post-transplant Vasculopathy

**Figure 15:**
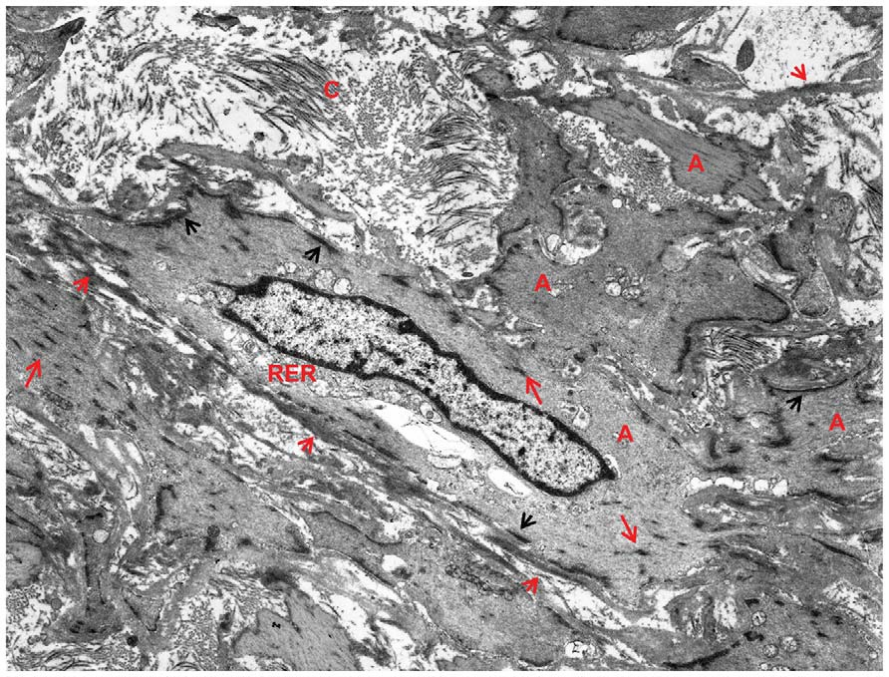
Explanted heart (transplant 2) with post-transplant CA vasculopathy showing similar features to those in KD LMP. In patient 30, the second transplanted heart was removed because of post-transplant vasculopathy; pathologic myofibroblasts were observed in the luminal lesions of the coronary arteries. This myofibroblast is similar to the one in Figure 10B with its broad irregular processes (right field). It has extensive dense plaques (short black arrows), abundant actin (A) and some dense bodies (long red arrows). External lamina is both attached and shed (short red arrows). The loose banded collagen (C) is in the upper left of the field. Case 30C, original magnification 4,000×.

One KD patient (case 30) underwent a total of three heart transplants, the first for ischemic KD coronary vasculopathy, and two more for ischemic post-transplant vasculopathy. The KD heart had thrombosed CAA, while the subsequent hearts had the CA stenosis typical of post-transplant vasculopathy, with some similarities to KD LMP. The CA media showed the same pattern of adventitial SMC entering the periphery of the media and SMC/myofibroblasts exiting the opposite surface and entering the luminal mass. TEM of coronary artery tissue retrieved from a paraffin block from the second transplanted heart revealed SMC-derived LMP-like myofibroblasts (Figure 15).

While the initial KD heart had typical SA/C vasculitis/damage of the CA media and SA/C-LMP, the CA of the two post-transplant vasculopathy hearts showed little to no chronic inflammation of the media or of their luminal lesions, mild medial and IEL damage, and both lipid-laden macrophages and SMC scattered throughout the lesions. However, the post-transplant vasculopathy lesions lacked the calcium, cholesterol clefts, atheromas, and multi-nucleated macrophages typical of atherosclerotic plaques.

### Non-vascular Pathology

A number of other intriguing, non-vascular observations were made; these are described in Table S1.

## Discussion

Our study reveals a three processes model of KD pathology newly described based on the English language KD literature and the availability of original figures in KD articles published in foreign journals (Table 3). Some of our observations appear to directly contrast with those previously described (Table 4). Our findings lead us to propose a range of clinicopathologic outcomes following infection with the etiologic agent of KD (Table 5).

**Table 3:** Newly described pathologic features observed in the 41 KD patients.

*Acute neutrophilic necrotizing arteritis* (NA) causes the saccular aneurysms that fatally rupture or thrombose within the first month after onset; survival can be followed by superimposed thrombotic episodes or progressive coronary insufficiency that can be fatal at any time.
NA involves medium sized muscular and elastic arteries but not veins, pulmonary arteries, and apparently not the aorta.
Severe *subacute/chronic (SA/C) vasculitis* can cause saccular aneurysms that can fatally thrombose (but apparently not rupture) in the first two months and thereafter; persistent SA/C may account for morbidity and mortality from worsening of CAA months and years after acute KD in some cases [67].
The intra-luminal stenosing lesions caused by *luminal myofibroblastic proliferation* (LMP) contain smooth muscle cell (SMC)-derived pathologic myofibroblasts that produce extracellular matrix (ECM) products in a background of SA/C inflammatory cells (SA/C-LMP).
LMP can also occur in areas remote from active SA/C inflammation, possibly due to circulating factors.
SA/C-LMP lesions do not undergo organization or re-canalization nor represent granulation/scar tissue.
Transformation of medial SMC into myofibroblasts has been hypothesized but it required the TEM performed in this study to be confirmed.
Adventitial SMC can apparently replace medial SMC and can also transform directly into myofibroblasts, when medial SMC are lacking.
SA/C and SA/C-LMP also involves veins, pulmonary arteries, and the aorta, but apparently only to a relatively mild, subclinical degree.
SA/C inflammatory cells are predominantly small lymphocytes accompanied by varying numbers of eosinophils and plasma cells, while macrophages, mast cells, and neutrophils are rare.
The majority of lesional macrophages are apparently recruited to areas of medial and adventitial SA/C damage, where they scavenge debris and then migrate to draining lymph nodes.
There appears to be no consistent pattern to the location, abundance, and extent of fusiform dilations, saccular aneurysms, and stenosing SA/C-LMP lesions among cases.
Calcification occurs within organizing and organized thrombi, but not in the remaining tissue/wall of CAA.
Infarcts of the kidneys, spleen, and adrenals due to SA/C-LMP are relatively common, but were apparently not clinically evident at the time of death/transplant.
KD patients can have an interstitial “eosinophilic-type” myocarditis accompanied by profound edema and interstitial lymphocytes, but without myocyte degeneration or necrosis.
Pseudo-myocarditis, from SA/C inflammation directly extending from CA pan-arteritis, is relatively common and can be misinterpreted as true myocarditis.
SA/C endocarditis appears to be a virtually universal phenomenon that can seriously involve the contiguous valves. The fibrotic endocardium frequently undergoes elastosis consistent with endocardial fibroelastosis.
Pericarditis varies in the degree of SA/C inflammation, which is usually most concentrated over inflamed CA.
Stenosing LMP with little to no SA/C inflammation and intact elastic laminae and media can occur at “remote” arterial sites, suggesting that factors from active SA/C inflammation can circulate and act widely.
Patterns of myocardial ischemia/infarction can be strikingly complex.
Patients appear capable of surviving surprisingly extensive myocardial scarring.
A spectrum of microscopic SA/C and lymphocyte/macrophage aortitis can be seen.
Eosinophilic and giant cell-like myocarditis may be associated with KD.
KD clinicopathology develops in roughly two phases: acute NA (likely the direct effect of a virus) and SA/C inflammation causing SA/C-LMP stenosis (likely immune-mediated).

**Table 4:** Pathologic observations that differ from those previously reported.

Neither granulomatous inflammation nor granulomas were detected.
Severe CAA do not “regress”, “resolve”, or “remodel”, but rather they thrombose.
Neutrophils characterize NA, while lymphocytes, plasma cells, and eosinophils characterize SA/C inflammation.
Small vessel pathology is rare and mild, and involves intra-myocardial and intra-renal arteries.
There was no medial SMC hyperplasia.
There was minimal medial fibrosis/scarring.
There were no histologic features of atherosclerosis even in remote deaths or transplants.

**Table 5:** Potential clinicopathologic outcomes following infection with the KD etiologic agent.

If the child lacks the “required” genetics, the ubiquitous seasonal infection will likely be essentially inconsequential, i.e., just another mild respiratory illness of childhood. In some of these individuals it may lead to ciliated bronchial cell intracytoplasmic inclusions. Whether these children harbor the putative KD agent for a prolonged period in an immunologically controlled/suppressed state is unknown.
Some genetically predisposed children may develop some or all of the characteristic clinical signs and symptoms of KD, but no overt vasculitis.
Other predisposed children may develop the clinical features of KD with inconsequential CA vasculitis.
Others may develop CA or NCA fusiform dilations with or without aneurysms that neither rupture nor thrombose, and develop asymptomatic SA/C and SA/C-LMP, which may progress slowly until clinically significant [68]. Echocardiography may underestimate the presence of critical SA/C-LMP CA pathology, i.e. “hidden” KD vasculopathy.
At about 3–4 weeks, a very small percentage of IVIG-untreated or resistant patients die from CAA and myocardial ischemia, ruptured CA or NCA aneurysm, or thrombosis of a NCA (Table 2).
Patients who do not die from an “acute” event can experience progressive LMP stenosis until they ultimately die from coronary insufficiency (Cases 25 at 4.5 months and 27 at 7.5 months) or receive a cardiac transplant (Cases 30 at 15 months, 32 at 18 months, and 33 at 2 years); the complicated, unpredictable nature of SA/C-LMP lesions makes coronary artery bypass grafting potentially difficult and angioplasty of variable benefit.
“Stable” CAA may eventually accumulate enough thrombotic material to eventually cause critical myocardial ischemia.

The fact that about two-thirds of our cases developed KD in the post-IVIG era (after the late 1980s) attests to the continued significance of this enigmatic disease. Although this study is by necessity a mortality and near-mortality transplant study, it raises the question of the scope of its undocumented long-term morbidity and mortality. Many of our patients were diagnosed after the 10th day of illness, and in some cases the diagnosis was not suspected until at or near the time of death. This emphasizes the continued critical need for a timely diagnostic test and improved treatment.

Considering the degree of arterial wall destruction due to NA and severe SA/C panarteritis, it seems counterintuitive that the resulting aneurysms could possibly “remodel”, “resolve”, or “regress” [33], [35], [36], [37], [38]. The aneurysmal arteries have lost both elastic laminae, most if not all of their media, and portions of their adventitia. The concept of “remodeling” or “regression” arises from a misinterpretation.Non-lethal thrombi variably fill aneurysmal cavities (the apparent lumina), giving the impression that the aneurysms are actually decreasing in size. Thus, shrinkage of focal or ring-shaped calcifications as assessed by CA radiography actually indicates progression of the intraluminal thrombotic process. Thus, the phenomenon of “arteries within the artery” is a consequence of organization and recanalization of thrombi [39]. Echocardiography primarily images the proximal portion of the CA and may therefore underestimate the extent of distal CA vasculopathy. Intravascular ultrasound studies using interpretation based on studies of atherosclerosis, a very different pathologic process from KD, must be interpreted with caution [40], [41] and should be correlated with actual KD vasculopathy specimens; for example, it is unlikely that LMP can be distinguished from organizing thrombi by intravascular ultrasound.

SA/C vasculitis is almost assuredly responsible for transition of SMC into myofibroblasts and the subsequent creation of LMP lesions. Transforming growth factor β stimulates transition of fibroblasts into classic wound healing myofibroblasts, but the factors that do so for SMC transition into LMP myofibroblasts are unknown but of great interest. Although they are ultrastructurally very similar, LMP myofibroblasts tend to be more pleomorphic and activated than those in wound healing. It is possible that LMP lesions represent a form of non-resolving wound healing; persistent inflammation associated with chronic, poorly healing wounds is well-documented [42]. Ultimately, healing is associated with myofibroblast apoptosis, which is not seen in LMP lesions. LMP lesions could actually be a response to the wounding of the media by SA/C inflammation, stimulating the SMC transition to myofibroblasts and proliferation of luminal mesenchymal/inflammatory lesions, as if the open lumen was being interpreted as a “defect”.

It is critical to know why the coronary arteries are always targeted by the etiologic agent of KD in patients who develop vasculitis, while other similar sized muscular and elastic arteries are only variably affected. Although other arterial diseases may involve the CA, none consistently attacks them. A likely possibility is that there exists a heterogeneity among endothelial cells from different body sites with regards to chemokines, adhesion molecules, or other markers. The receptors for the KD agent may be more prevalent on CA endothelium [43]. The intracranial vessels are spared, while the aorta and pulmonary arteries only undergo a minimal amount of pathology. This extraordinary CA specificity is not observed in any other vascular disease, including atherosclerosis, which involves one or more of the three major arterial systems: CA, aorta, and central nervous system. KD may serve as a model for other pathologies, especially other occlusive vasculitides, and also for basic biologic processes.

Interstitial myocarditis has been previously reported as an acute and subacute/chronic phenomenon in KD and may be responsible for the cardiac dysfunction commonly observed during the acute illness, which is generally responsive to IVIG [44], [45], [46]. It has been difficult to correlate the clinical diagnosis with myocardial pathology, since there have been few published autopsies from early in the illness. A single report of a child who died 9 days after disease onset, presumably from an arrhythmia, showed myocardial edema with neutrophils and lymphocytes infiltrating from intramural micro-vessels and small arteries, but there was no mention of myocyte damage [20]. Vascular damage may be the cause of the edema. Some of the reported myocardial inflammation may actually represent “pseudo-myocarditis” from spillover of SA/C pan/peri-arteritis of adjacent coronary arteries [47]. Normal serum cardiac troponin levels in the acute phase of KD suggest that myocyte necrosis is not significant [48]. Our day 10 “eosinophilic-type” myocarditis fatality is both curious and seemingly unique for KD. It remains to be determined whether the two conditions are related to the same etiologic agent. It is unclear whether the “myocarditis” seen in endocardial biopsies months to years after KD onset might either be due to ischemia or endocarditis [49].

While KD is rare in healthy adults, it may be more prevalent in immunocompromised individuals, e.g. human immunodeficiency virus/acquired immunodeficiency syndrome (HIV/AIDS) [50], [51], [52], [53], [54], [55]. At least 20 HIV/AIDS adult patients (average age of38 years) (CD4 lymphocyte counts less than 200/mm^3^ in 17 patients and most with high viral loads) have been reported as having presented with KD-like clinical signs and symptoms, although none apparently developed documented CA pathology [50]. Recurrence of KD symptoms was reported in 5 of the 20 patients. A child with HIV infection who developed myocardial infarction due to thrombosis of a right coronary artery aneurysm was has been reported [56]. If HIV/AIDS does increase the risk of developing KD, could any immunodeficient patient experience reactivation of an immunologically sequestered KD agent that was associated with a previous asymptomatic or symptomatic childhood infection that persisted dormantly? Alternatively, could immunodeficiency modify the genetic influences on KD susceptibility?

Cells identified as “myofibroblasts” (based only on IHC for SMA) in animal models of coronary injury and following coronary angioplasty and intracoronary stent placement have been considered to derive from adventitial fibroblasts. Medial SMC, endothelial cells, circulating bone marrow-derived fibrocytes, pericytes, and mesenchymal stem cells have also been proposed as possible sources [57], [58], [59]. In a rat carotid artery injury model, the cell of origin was considered to be an adventitial myofibroblast [60]. However, a recent study using bromodeoxyuridine tracing to identify the neo-intimal cells in a swine balloon angioplasty model concluded that they are of SMC origin [61]. Since atherosclerosis is considered to be an inflammatory process with medial SMC [62] and even myofibroblasts [63] as important plaque components, correlative LM, IHC, and TEM studies are necessary to determine if these cells are truly related to the classic myofibroblast of wound healing, and if there is a relationship to SA/C inflammation and SA/C-LMP vasculopathy.

Previous LM studies of post-transplant vasculopathy (PTV) concluded that both proliferating SMC and “myofibroblasts” comprise its neo-intima [64]. It was of great interest to be able to study the KD heart and two subsequent transplanted hearts from the same patient. There were both similarities and differences between the PTV and KD vasculopathy. The TEM of the PTV CA revealed myofibroblasts identical to those in KD LMP, as well as the same “in-and-out” pattern of adventitial SMC entry to the media and transition of medial SMC into myofibroblasts. However, PTV differed from KD in its lack of SA/C inflammation, its virtual preservation of the media, the more fibrotic and less cellular obstruction, and luminal lesions that contained both foamy macrophages and SMC. These observations suggest the possibility that pathologic myofibroblasts may not be restricted to the occlusive vasculopathy of KD.

A number of potentially important clinical implications derive from this study (Table 6). Employing the three process model for KD vasculopathy greatly facilitated our ability to perform clinical:pathologic and radiographic:pathologic correlation, both for our cases and published cases. We believe that a key to advancing our understanding of this enigmatic disease is to establish a multi-functional “Kawasaki Disease Center” to collect clinical, pathologic, laboratory, and radiographic data from as many diagnosed patients as possible; collect tissue and fluid specimens for study, teaching, and research; provide timely diagnostic and clinical assistance; and offer educational opportunities for clinicians, pathologists, and researchers. Establishing a full time “Registry, Repository, Diagnostic, and Educational Center” seems a worthy objective. Thorough gross and microscopic autopsy and transplanted heart examination using specifically designed protocols with extensive digital camera documentation is critical. All CA and medium-sized NCA must be “bread-loafed” for both gross and microscopic examination since mural and luminal vasculopathy due to SA/C, SA/C-LMP, and LMP can involve vessels that appear normal on gross examination. Clinically significant SA/C, LMP, and SA/C-LMP lesions can be observed in persistent and recoiled fusiform dilations [65]. Representative normal and pathologic specimens fixed for light and transmission electron microscopy as well as appropriately frozen specimens are critical. Careful examination of specimens from deaths of individuals with documented histories of KD, who apparently did not die from the disease is of enormous importance. Likewise is the study of specimens from individuals without a known KD history, but who die from CA pathology that is not typical of atherosclerosis, such as thrombosed or ruptured CAA or non-atheromatous-type luminal stenosis [66]. The younger the individual, the greater the possibility that the individual might have had undiagnosed KD.

**Table 6:** Clinical implications of this study.

IVIG treatment is likely to abrogate NA pathology and thus affect subsequent events, so early therapy is likely critical.
Since the threat of new and the extension of previous CA thrombi persists, more aggressive and prolonged anti-platelet and anti-thrombotic therapies for patients with significant CA abnormalities may be warranted.
A study of prolonged anti-inflammatory therapy in patients with CA abnormalities should be considered, since persistent inflammation is a feature of SA/C and SA/C-LMP.
Identification of therapies that interfere with the transition of SMC to myofibroblasts and progression of SA/C-LMP and LMP lesions is urgent.
Luminal narrowing from CAA thrombosis followed by calcification of organizing thrombi should aid with pathologic/radiographic interpretation.
Apparently “regressed” fusiform dilations are still capable of undergoing luminal narrowing by persistent SA/C and SA/C-LMP.
Endocarditis appears to be very common and can potentially cause serious valvular disease.
Endocarditis often leads to endocardial fibroelastosis, which may have a restrictive cardiac effect and requires monitoring.
Long-term survivors with significant NCA pathology may ultimately suffer some pancreatic, renal, or adrenal dysfunction.
KD vasculopathy does not appear to progress to atherosclerotic coronary artery disease.

It is critical that the KD etiologic agent be identified, isolated, and propagated, so that we can understand the pathogenesis, derive a rapid diagnostic test, identify effective acute and chronic therapy, and make prevention possible.

## Supporting Information

Table S1 **Non-vascular pathology observed in the KD patients.**
(DOCX)
