# Supplementary material for: Three Linked Vasculopathic Processes Characterize Kawasaki Disease: A Light and Transmission Electron Microscopic Study
Source: PLoS One. 2012 Jun 18;7(6):e38998. doi: 10.1371/journal.pone.0038998 (PMC3377625; doi:10.1371/journal.pone.0038998)
Supplement: Table S1 — Non-vascular pathology observed in the KD patients. (DOCX) [file pone.0038998.s001.docx]

**Table S1. Non-vascular pathology observed in the KD patients.**

- 1. Erythrophagocytosis by sinus macrophages of lymph nodes of the neck and elsewhere, e.g., abdomen.
  2. Lipofuscin (secondary lysosomes ; “aging pigment”), which is characteristic of aging and cachexia, was extremely abundant in cardiac myocytes and hepatocytes.
  3. Cells with the pathognomonic “caterpillar” (longitudinally sectioned) and “owl-eye” (cross sectioned) chromatin pattern classic for Anitschkow cells were present in every heart, often in striking quantities, as well as in some extra-cardiac squamous cells and fibroblasts. In addition to being present in cardiac myocytes and macrophages, the chromatin pattern was seen in cardiac endothelial cells, fibroblasts, and Schwann cells, none of which have apparently been previously reported to have Anitschkow chromatin.
  4. SA/C-inflamed epicardial nerves.
  5. Mitochondrial calcification of random “healthy” myocytes of otherwise ischemic hearts.
  6. Dystrophic calcification of healing myocardial infarcts.
  7. Random irregular foci of soft tissue SA/C inflammation containing mesenchymal cells and quiescent macrophages in several cases.
  8. Variable degrees of SA/C inflammation/damage of the secretory ducts of pancreas, biliary tract, salivary glands, and trachea, which could be associated with varying degrees of pancreatitis and sialadenitis.
  9. Adrenal medullary necrosis associated with macrophages.
  10. Individual KD cases had the following: 1) ubiquitous foci of calcification in a child with normal electrolytes and renal function and 2) generalized pigmented mononuclear and multinucleated macrophages.
